# Acenaphthoquinoxaline Derivatives as Dental Photoinitiators of Acrylates Polymerization

**DOI:** 10.3390/ma14174881

**Published:** 2021-08-27

**Authors:** Ilona Pyszka, Beata Jędrzejewska

**Affiliations:** Faculty of Chemical Technology and Engineering, UTP University of Science and Technology, Seminaryjna 3, 85-326 Bydgoszcz, Poland

**Keywords:** acenaphthoquinoxaline derivatives, photoinitiators, photoinduced electron transfer process, radical polymerization

## Abstract

A series of dyes based on the acenaphthoquinoxaline skeleton was synthesized. Their structure was modified by introducing electron-withdrawing and electron-donating groups, increasing the number of conjugated double bonds and the number and position of nitrogen atoms, as well as the arrangement of aromatic rings (linear or angular). The dyes were investigated as a component in the photoinitiating systems of radical polymerization for a potential application in dentistry. They acted as the primary absorber of visible light and the acceptor of an electron, which was generated from a second component being an electron donor. Thus, the radicals were generated by the photoinduced intermolecular electron transfer (PET) process. Electron donors used differed in the type of heteroatom, i.e., O, S and N and the number and position of methoxy substituents. To test the ability to initiate the polymerization reaction by photoinduced hydrogen atom transfer, we used 2-mercaptobenzoxazole as a co-initiator. The effectiveness of the photoinitiating systems clearly depends on both the modified acenaphthoquinocaline structure and the type of co-initiator. The lower amount of heat released during the chain reaction and the polymerization rate comparable to this achieved for the photoinitiator traditionally used in dentistry (camphorquinone) indicates that the studied dyes may be valuable in this field.

## 1. Introduction

The most versatile and easily implemented polymerization method is radical polymerization. Free radicals are formed by a number of different mechanisms. They can be generated directly from an initiator that undergoes thermal or photolytic dissociation or may be formed in bimolecular processes following a photoinduced intermolecular electron (or proton) transfer [1,2,3].

A large number of organic compounds are used as polymerization photoinitiators [4,5,6]. Generally, they can be classified into Norish type I and II initiators [7]. The former one is typically aromatic carbonyl compounds with suitable substituents that facilitate homolytic photodissociation. Depending on the type and position of the functional groups, the unimolecular fragmentation of the precursor occurs within the bond located at position α (α-photodissociating initiators) [8,9,10,11] or β (β-photodissociating initiators) in relation to the carbonyl group [12]. This group of photoinitiators also includes organic compounds with weak O–O, S–S, N–S and C–N bonds in the molecule [13,14,15,16].

The type II photoinitiators are typically two-component photoinitiating systems consisting of a light absorber (photosensitizer, PS) and a co-initiator (an electron donor or a hydrogen atom donor) that generate radicals in bimolecular processes. They usually contain ketones, aldehydes, quinones and selected dyes in combination with a tertiary amine, carboxylic acids, borate salts and others electron donors that form photoredox pairs. Radicals, which start a chain reaction, are generated from a co-initiator according to photoinduced hydrogen atom transfer [17,18,19] or electron transfer [6,20,21,22,23,24,25,26,27,28,29,30,31] mechanism.

**Scheme 1:**
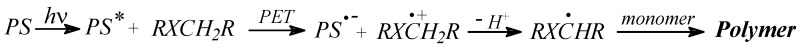
The processes that may occur during radical photoinduced polymerization initiated by the photoinduced intermolecular electron transfer (*PET*) mechanism; *PS* denotes photosensitizer, RXCH_2_R is an electron donor such as amine, sulfide, cyclosulfide-trithian.

Scheme 1 summarizes possible processes which may occur during the radical photoinitiated polymerization via the photoinduced intermolecular electron transfer process (PET) in the presence of electron donors such as aromatic amines or sulfides [2,6,20,21,32]. The initial step of the process involves one-electron transfer from the electron donor to the singlet (or triplet) state of the photosensitizer (*PS*). As a result, reactive intermediates, e.g., a *PS* radical anion and an electron donor radical cation, are formed. Subsequent hydrogen abstraction leads to radicals that are capable of initiating polymerization.

**Scheme 2:**
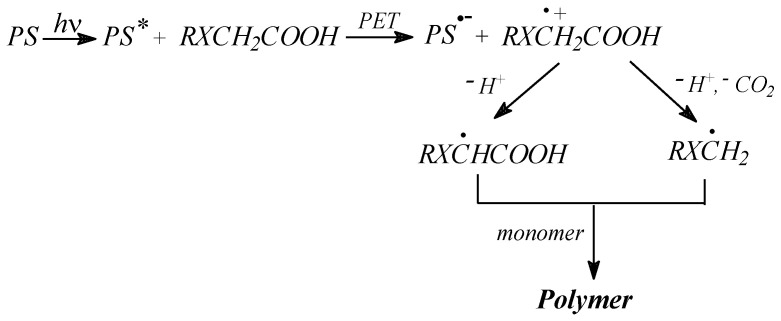
The processes that may occur during radical photoinduced polymerization initiated by the photoinduced intermolecular electron transfer (*PET*) mechanism followed by proton abstraction or decarboxylation; *PS* denotes photosensitizer, *RXCH*_2_*COOH* is an electron donor such as phenoxyacetic acid, thiophenoxyacetic acid, N-phenylglycine, N-phenyliminodiacetic acid.

Another important mechanism of polymerization initiation involves a photoinduced intermolecular electron transfer (PET) process followed by proton abstraction and decarboxylation (Scheme 2). The commonly used co-initiators in this case are phenoxyacetic acid, thiophenoxyacetic acid, N-phenylglycine, and N-phenyliminodiacetic acid. The electron transfer from the acid to the singlet (or triplet) state of the sensitizer is followed by:

1. diffusion of the radical-ion pair.
2. intramolecular proton transfer of the radical-ion pair that is retained in the solvent cage.

In polar solvents, the first process is more efficient and leads to the decarboxylation of a carboxylate radical and formation of an alkyl radical. In less polar and viscous solvents, there is no separation of the radical ions and a proton transfer to produce a radical centered on α-carbon with respect to the carboxylate group that takes place. Thus, two different radicals produced from the co-initiator in the secondary reaction can react with the monomer to initiate polymerization [21].

**Scheme 3:**
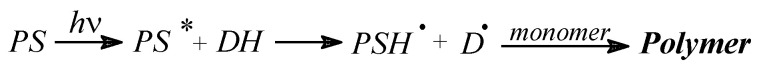
The processes that may occur during radical photoinduced polymerization initiated by the photoinduced intermolecular hydrogen atom transfer mechanism; *PS* denotes photosensitizer, DH is a hydrogen donor, e.g., 2-mercaptobenzimidazole.

The mechanism of initiation of the polymerization reaction may also be based on the hydrogen atom transfer process. The photosensitizer abstracts a hydrogen atom from a co-initiator, forming two radicals. These radicals can then react with monomer molecules to start a chain reaction. Most commonly used hydrogen donors are amines (Scheme 3).

Photoinduced radical polymerization of various monomers has been gaining more and more interest in various industries in recent decades [6,32]. Many of the developed photoinitiating systems meet the requirements for specific applications in the technology of protective coatings [33,34,35], graphics industry [36], microelectronics [37], optical disc production [38], holography [39,40,41], stereolithography [42,43,44] and medicine [45,46,47,48,49,50,51]. Photopolymerization has also become an integral part of dental practice [52]. It is especially used in in-vivo production of dental composites [53]. 

Applying photochemically initiated polymerization for obtaining dental polymer composites is connected with its unique properties, such as a short time of monomer/filler compositions curing (high polymerization rates in fractions of seconds, resulting from the rapid formation of radicals), low energy consumption, conducting the polyreaction at ambient temperature, and spatial resolution (polymerization only in irradiated areas). The main limitation of the method is the thickness of the cured layer due to the absorption of light when passing through the irradiated layer. 

The polymer composite filling materials typically consist of:

- monomers and polyfunctional monomers, e.g., trimethylolpropane triacrylate (TMPTA), pentaerythritol triacrylate (PETA), 1,6-hexanediol diacrylate (HDDA), bisphenol A diacrylate (bis DMA);
- a photoinitiator, e.g., camphorquinone;
- a co-initiator, usually aromatic amines, e.g., *N*,*N*-dimethyl-*p*-toluidine;
- reinforcing fillers, e.g., quartz, silicate glass, silicon nitrides, etc., coated with 3-(trimethoxysilanyl)propan-1-ol acrylate. This additive is used to improve the adhesion of the filler to the polymer matrix;
- inhibitors that prevent premature polymerization during storage;
- light stabilizers that prevent the filling from changing color;
- compounds that allow to match the color of the filling to the natural color of the patient’s teeth.

A typical photocuring dental composition comprises about 10%–30 wt.%. of organic components and ca. 70 wt.% of inorganic fillers. The most popular materials for obtaining dental composites by photopolymerization are (meth)acrylate monomers characterized by high reactivity. In addition, the composition contains photoinitiators that absorb blue light emitted by dental lamps and a co-initiator that accelerates the polymerization process [53]. The use of UV radiation for in-vivo photocuring of dental composites is not recommended due to the safety of dental treatments. It can interact with tissues, contributing to the development of cancer or cause tissue burns. Moreover, (meth)acrylate monomers can cause allergic reactions while aromatic amines used as co-initiators can be carcinogenic and mutagenic, except that dental composites based on cross-linking monomers show the problem of 2–14% volumetric shrinkage during the photopolymerization process [54,55]. 

The polymerization shrinkage causes mechanical stresses in the filling, which significantly reduces the mechanical strength. As a consequence, defects may arise in the composite-tooth bond, which lead to bond failure, microleakage, postoperative sensitivity, and recurrent caries. Therefore, various types of fillers are added to the dental composite, which are able to reduce the polymerization shrinkage to 1–3% and at the same time increase the mechanical strength of the filling (to abrasion, crushing and washing out). However, a large amount of filler (up to 70%) limits the depth of light penetration into the polymerization layer and the monomer does not react completely. Therefore, in-vivo photopolymerization must be carried out in thin layers, the thickness of which should not exceed 1 mm. 

Despite significant progress in development of new photoinitiating systems for dental composites, they still have several significant disadvantages mentioned above. Therefore, research conducted around the world is aimed at developing dental compositions that would provide durable fillings and would be completely safe for humans. This can be achieved by eliminating from the dental composition’s cytotoxic amines and acrylate monomers as well as camphorquinone which causes the yellow staining of the fillings. In addition, the mechanical properties of the fillings should be improved, and the polymerization shrinkage reduced. It should also be borne in mind that visible light must be used in the curing process instead of harmful UV radiation.

The above-mentioned aspects and the progress in the applied technologies mean that new photoinitiating systems based on different photosensitizers and co-initiators with improved properties are still being sought.

Therefore, in this work we focused on modifying the structure of dyes based on acenaphthoquinoxaline skeleton to obtain new colorless sensitizers operating in the visible light region for an application in dentistry. To eliminate the mutagenic aromatic amines from the photocurable composition, we used organic acids as co-initiators. The studies on the effectiveness of the acid sensitization by acenaphthoquinoxaline derivatives were conducted upon visible light irradiation using microcalorimetric method. The photoinitiating ability of the tested photoredox pairs were compared with a camphorquinone, a typical photoinitiator used in dentistry. Finally, the photopolymrization experiments were repeated in the presence of dental filler, i.e., glass ionomer in order to check the photoinitiating ability of the tested systems to obtain dental composites by radical photopolymerization. The glass ionomers in dentistry are used as: underlay material, temporary filling material, final filling material or sealing material [56,57].

## 2. Materials and Methods

### 2.1. Reagents

Reagents for the synthesis of the acenaphthoquinoxaline derivatives were purchased from Alfa Aesar Co., Ward Hill, MA, USA (acenaphthoquinone, 1,2-diaminobenzene), Sigma-Aldrich Co., Saint Louis, MI, USA (2,3-diaminopyridine, 2,3-diaminonaphthalene, 4-methoxy-1,2-diaminobenzene, 9,10-diaminophenanthrene), Acros Organics Co., Pittsburgh, PA, USA (3,4-diaminobenzoic acid, 1,2-diammonocyclohexane) and Fluorochem Co., Hadfield, UK (2,3-diaminophenazine). Solvents for the synthesis and spectroscopic measurements, i.e., acetic acid, ethyl acetate, 1-methyl-2-pyrrolidone (MP), chloroform (CHCl_3_) and deuterate chloroform (CDCl_3_), dimethylsulfoxide (DMSO-d_6_) were supplied by Sigma-Aldrich Co., Saint Louis, MI, USA.

Monomer, trimethylolpropane triacrylate (TMPTA), a commercial photoinitiator, camphorquinone (CQ), and co-initiators, i.e., 2-mercaptobenzoxazole (MBX), (phenyltio)acetic acid (PhTAA), phenoxyacetic acid (PhAA), 4-methoxyphenoxyacetic acid (MPhAA), (3,4-dimethoxyphenylthio)acetic acid (diMPhTAA) were obtained from Sigma-Aldrich Co., Saint Louis, MI, USA, whereas *N*-phenyliminodiacetic acid (PhIDAA) was from Lancaster, Spartanburg, United States. Dental material—glass ionomer Ketac Fil Plus powder was purchased from the 3M-ESPE Co., Maplewood, NJ, USA. 

Thin layer chromatography plates (DC-Plastikfolien Silica gel 60 F254, 0.2 mm) were from Merck Co., Kenilworth, UK. 

**Scheme 4:**
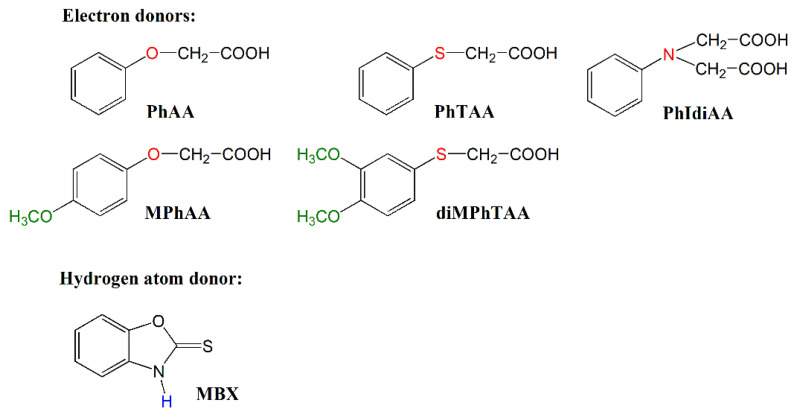
The structure of the co-initiators used in the photopolymerizing composition.

The structures of the co-initiators used are shown in Scheme 4. They are divided into two groups, i.e., electron donors and a hydrogen atom donor. 

### 2.2. General Procedure for Synthesis of Acenaphthoquinoxaline Derivatives 

**Scheme 5:**
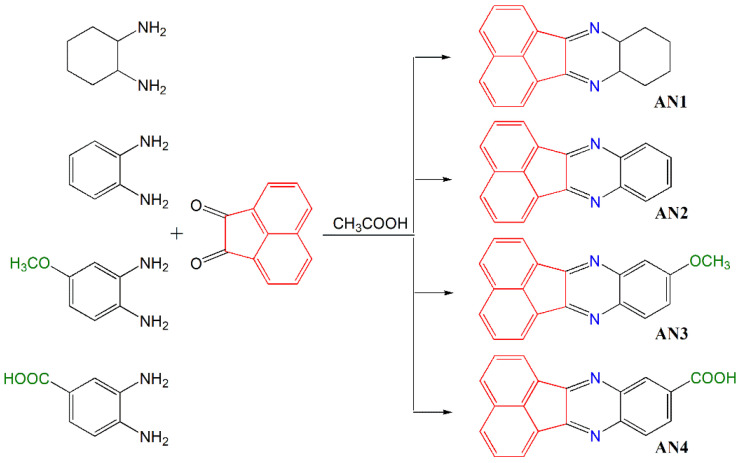
Synthesis routes of acenaquinoxaline derivatives (AN1–AN4) differing in the number of conjugated double bonds and the type of the substituent.

**Scheme 6:**
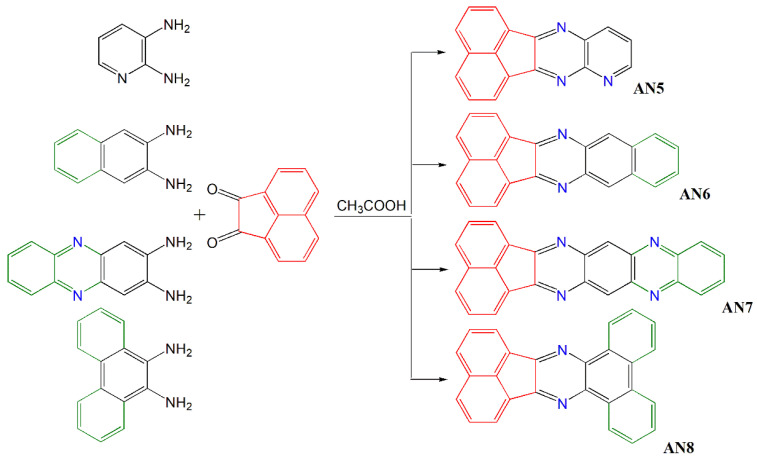
Synthesis routes of acenaquinoxaline derivatives (AN5–AN8) differing in the arrangement of aromatic rings (linear or angular) and the number and position of nitrogen atoms in the molecule.

The target molecules were synthesized by one-step reaction shown in Scheme 5 and Scheme 6. The acenaphthoquinoxaline derivatives (AN1–AN8) were obtained by refluxing (ca. 3 h) equimolar amounts of the corresponding diamines with acenaphthoquinone in glacial acetic acid (40–60 mL) according to the method described in the literature [58,59,60]. The progress of the reaction was monitored by thin layer chromatography on silica gel 60F 254 using chloroform as eluent. The precipitate formed after cooling down the reaction mixture was filtered and then recrystallized from ethyl acetate to afford (about 70–80%) the desired compound, usually pale (or light) yellow solid. The products were identified spectroscopically. The ^1^H and ^13^C NMR spectra of all synthesized compounds are presented in the ESI file. The structures of the synthesized chromophores (photosensitizers) are presented in Scheme 5 and Scheme 6.

- 8,9,10,11-Tetrahydroacenaphto[1,2-b]quinoxaline—AN1

The compound was prepared using acenaphthequinone (1.0 g, 5.5 mmol) and 1,2-diaminocyclohexane (0.626 g, 5.5 mmol) in 40 ml of glacial acetic acid; reflux 3 h. Obtained 0.5 g (71%) of light-yellow crystals, C_18_H_14_N_2_, 258.32 g/mol, m.p. 197–199 °C.

^1^HNMR (CDCl_3_): 8.15–8.13 (d, ^3^J_H,H_ = 8.0 Hz, 2H), 8.05–8.03 (d, ^3^J_H,H_ = 8.0 Hz, 2H), 7.78–7.75 (t, 2H), 7.61–7.59 (d, ^3^J_H,H_ = 8.0 Hz, 2H), 7.16–7.12 (t, 2H), 6.84–6.82 (d, ^3^J_H,H_ = 8.0 Hz, 2H), 4.87 (s, 2H).

^13^CNMR (CDCl_3_): 136.4, 135.0, 133.7, 131.8, 131.4, 130.7, 130.5, 128.2, 128.1, 128.0, 124.6, 124.2, 40.4, 40.2, 40.0, 39.8, 39.6.

- Acenaphto[1,2-b]quinoxaline—AN2

The compound was prepared using acenaphthequinone (1.0 g, 5.5 mmol) and 1,2-diaminobenzene (0.593 g, 5.5 mmol) in 80 ml of glacial acetic acid, reflux 3 h. Obtained 1.1 g (79%) of cream crystals, C_18_H_10_N_2_, 254.29 g/mol, m.p. 238–240 °C.

^1^HNMR (CDCl_3_): 8.71–8.69 (d, ^3^J_H,H_ = 8.0 Hz, 2H), 8.43–8.40 (m, 2H), 8.24–8.22 (d, ^3^J_H,H_ = 8.0 Hz, 2H), 7.96–7.92 (t, 2H), 7.90–7.87 (m, 2H).

^13^CNMR (CDCl_3_): 153.5, 140.5, 136.6, 131.1, 129.9, 129.9, 129.6, 129.1, 128.8, 122.7

- 9-Methoxyacenaphto[1,2-b]quinoxaline—AN3

The compound was prepared using acenaphthequinone (1.0 g, 5.5 mmol) and 4-methoxy-1,2-diaminobenzene (1.15 g, 5.5 mmol) in 50 ml of glacial acetic acid, reflux 3 h. Obtained 1.4 g (89%) of yellow crystals, C_19_H_12_N_2_O, 284.31 g/mol, m.p.195–196 °C.

^1^HNMR (CDCl_3_): 8.36–8.34 (d, ^3^J_H,H_ = 8.0 Hz, 1H), 8.32–8.30 (d, ^3^J_H,H_ = 8.0 Hz, 1H), 8.05–8.00 (m, 3H), 7.79–7.75 (m, 2H), 7.49–7.47 (d, ^3^J_H,H_ = 8.0 Hz, 1H), 7.36–7.33 (m, 1H), 3.95 (s, 3H, OCH_3_).

^13^CNMR (CDCl_3_): 161.3, 151.3, 136.4, 136.0, 130.9, 130.7, 130.0, 129.9, 129.8, 129.7, 129.4, 128.9, 128.6, 128.6, 128.4, 124.1, 122.8, 122.7, 56.1.

- Acenaphto[1,2-b]quinoxaline-9-carboxylic acid—AN4

The compound was prepared using acenaphthequinone (1.0 g, 5.5 mmol) and 3,4-diaminobenzoic acid (0.834 g, 5.5 mmol) in 80 ml of glacial acetic acid, reflux 0.5 h. Obtained 1.12 g (80%) of cream crystals, C_19_H_10_N_2_O_2_, 298.30 g/mol, m.p. 212–215 °C.

^1^HNMR (CDCl_3_): 13.46 (COOH, 1H), 8.72 (s, 1H), 8.50–8.48 (d, ^3^J_H,H_ = 8.0 Hz, 2H), 8.38–8.35 (m, 2H), 8.30 (s, 2H), 8.01–7.97 (t, 2H).

^13^CNMR (CDCl_3_):167.1, 155.3, 154.7, 143.3, 140.4, 136.6, 131.9, 131.5, 131.1, 131.1, 131.0, 130.7, 130.2, 130.2, 129.6, 129.4, 123.1, 123.0.

- Acenaphto[1,2-b]pirydo[2,3-e]pyrazine—AN5

The compound was prepared using acenaphthequinone (1.0 g, 5.5 mmol) and 2,3-diaminopyridine (0.599 g, 5.5 mmol) in 40 ml of glacial acetic acid, reflux 2 hours. Obtained 1.22 g (75%) of yellow crystals, C_17_H_9_N_3_, 255.27 g/mol, m.p. 223–225 °C.

^1^HNMR (CDCl_3_): 9.20 (d, 1H), 8.75–8.73 (d, ^3^J_H,H_ = 8.0 Hz, 1H), 8.64–8.62 (d, ^3^J_H,H_ = 8.0 Hz, 1H), 9.51–8.49 (d, ^3^J_H,H_ = 8.0 Hz, 1H), 8.25–8.22 (m, 2H), 7.97–7.91 (m, 2H), 7.86–7.83 (m, 1H).

^13^CNMR (CDCl_3_): 157.1, 154.9, 152.3, 150.5, 138.4, 137.2, 136.4, 131.1, 130.8, 130.2, 130.0, 129.9, 128.9, 128.6, 124.2, 123.3, 122.3.

- Acenaphto[1,2-b]benzo[g]quinoxaline—AN6

The compound was prepared using acenaphthequinone (1.0 g, 5.5 mmol) and 2-aminonaphthalene (0.87 g, 5.5 mmol) in 60 ml of glacial acetic acid, reflux 2 hours. Obtained 1.18 g (70%) of yellow crystals, C_22_H_12_N_2_, 304.35 g/mol, m.p. 353–354 °C.

^1^HNMR (CDCl_3_): 8.72 (s, 2H), 8.44–8.42 (d, ^3^J_H,H_ = 8.0 Hz, 2H), 8.09–8.07 (m, 4H), 7.84–7.80 (m, 2H), 7.56–7.52 (m, 2H).

^13^CNMR (CDCl_3_): 138.3, 135.2, 134.2, 133.7, 130.9, 130.7, 130.3, 130.2, 130.1, 129.9, 129.7, 129.4, 129.0, 128.8, 128.8, 128.6, 128.5, 127.8, 127.4, 127.1, 127.1, 127.0.

- Acenaphto[1′,2′:5,6]pyrazine[2,3-b]phenazine—AN7

The compound was prepared using acenaphthequinone (1.0 g, 5.5 mmol) and 2,3-diaminophenzine (1.16 g, 5.5 mmol) in 60 ml of glacial acetic acid, reflux 2 hours. Obtained 1.23 g (87%) of yellow crystals, C_24_H_12_N_4_, 256.38 g/mol, m.p. 265–268 °C.

^1^HNMR (CDCl_3_): 8.69–8.67 (d, ^3^J_H,H_ = 8.0 Hz, 2H), 8.50–8.48 (d, ^3^J_H,H_ = 8.0 Hz, 2H), 8.28–8.25 (d, ^3^J_H,H_ = 12Hz, 2H), 8.00–7.96 (m, 4H), 7.40 (s, 1H), 7.0 (s, 1H).

- Dibenzoacenaphto[1,2-b]quinoxaline—AN8

The compound was prepared using acenaphthequinone (1.0 g, 5.5 mmol) and 9,10-diaminophenanthene (1.14 g, 5.5 mmol) in 60 ml of glacial acetic acid, reflux 0.5 h. Obtained 1.60 g (82%) of yellow crystals, C_26_H_14_N_2_, 354.41 g/mol, m.p. 284–285 °C.

^1^HNMR (CDCl_3_): 9.46–9.43 (t, 1H), 8.95-8.93 (t,1H), 8.61–8.59 (d, ^3^J_H,H_ = 8.0 Hz, 1H), 8.46–8.44 (d, ^3^J_H,H_ = 8.0 Hz, 1H), 8.38–8.36 (d, ^3^J_H,H_ = 8.0 Hz, 1H), 8.1 (m, 3H), 8.1–8.01 (m, 2H), 7.94–7.91 (m, 4H).

### 2.3. Methods

Nuclear magnetic resonance (NMR) spectra were recorded on a Bruker Ascend™ 400 NMR spectrophotometer, Billerica, United States in deuterated dimethylsulfoxide (DMSO-d_6_) or deuterated chloroform (CDCl_3_).

Electronic absorption spectra were recorded on a Shimadzu UV-1280 spectrophotometer, Kioto, Japan. Steady-state fluorescence and phosphorescence spectra were performed on a Hitachi F-7100 spectrometer, Tokio, Japan. The fluorescence measurements were carried out at room temperature whereas the phosphorescence spectra were obtained at a liquid nitrogen temperature. All experiments were performed in an anhydrous ethanol (EtOH).

The measurements of both the oxidation and reduction potentials of the synthesized dyes and electron donors were made in a 0.1 M tetrabutylammonium perchlorate in anhydrous acetonitrile using an Electroanalytical MTM System (Krakow) Model EA9C-4z, EA, Krakow, Poland. A platinum 1 mm disk electrode was used as the working electrode, a Pt wire constituted the counter electrode, and Ag-AgCl electrode served as a reference electrode.

The kinetics of photoinitiated polymerization were investigated by a microcalorimetric method [29] recording changes in the heat evolution at constant time intervals (every 1 s) during polymerization. The thermo-sensitive diode was used as a temperature sensor. A Cromalux 75 dental lamp, Mega Physik, Rastatt, Germany with a beam intensity of 30 mW was applied as the light source. Its intensity was checked using a Field Master meter by Coherent. The distance of the sample from the light source was the same for all measurements. Each measurement was repeated at least three times.

The photopolymerization compositions consisted of 0.9 g of TMPTA, 0.1 mL of MP, photosensitizer and co-initiator. The concentration of the synthesized compounds (photosensitizers) varies from 8.0 × 10^−2^ mol dm^−3^ to 2.3 × 10^−4^ mol dm^−3^ depending on their molar absorption coefficient. The concentration of the co-initiators was 0.1 mol dm^−3^.

In order to compare the initiating efficiency of the polymerization reaction by the tested systems, a commercial photoinitiator, camphorquinone (CQ), was also used. Its concentration was 0.675 mol dm^−3^. 

The polymerization process with the addition of glass ionomer was carried out in a Teflon ring with a diameter of 10 mm and a thickness of 3 mm. The ratio of monomer composite to fillers was 3:7. The photopolymerization time was 30 s. The incident light of the dental lamp covered the entire surface of the ring. Its intensity was 30 mW/cm^2^.

## 3. Results

### 3.1. Molecular Design and Synthetic Procedures

The structures of acenaphthoquinoxaline derivatives have been modified to adjust their spectroscopic properties, especially absorption properties, so as to obtain compounds suitable to effectively initiate polymerization upon visible light irradiation. They differ in the number of conjugated double bonds as well as the type of substituent, i.e., an electron acceptor (–COOH) and electron donor (–OCH_3_) groups. The modification was also aimed at obtaining dyes with a different arrangement of aromatic rings (linear or angular) and the number and position of nitrogen atoms in the molecule. 

The acenaphthoquinoxaline (AN1–AN8) derivatives were synthesized by the condensation of the appropriate diamines with acenaphthoquinone in glacial acetic acid according to the method described in literature [58,59,60] and shown in Scheme 5 and Scheme 6. The structure and purity of the dyes were verified by NMR spectroscopy. The lack of an amino group signal in the ^1^H NMR (at 3.0–5.0 ppm) spectra confirmed the formation of a condensation product with a characteristic –C=N– bond (signal at 150.0–160.0 ppm in the ^13^C NMR spectra). The presence of signals from all protons and carbons in the NMR spectra proves that the dyes have the desired structure. All synthesized compounds (AN1–AN8) were obtained as light yellow or cream crystals, which also showed a pale-yellow color upon dissolution. This is very important considering their potential use in dentistry, as it allows you to obtain natural shades of fillings.

### 3.2. Spectroscopic Properties

**Figure 1:**
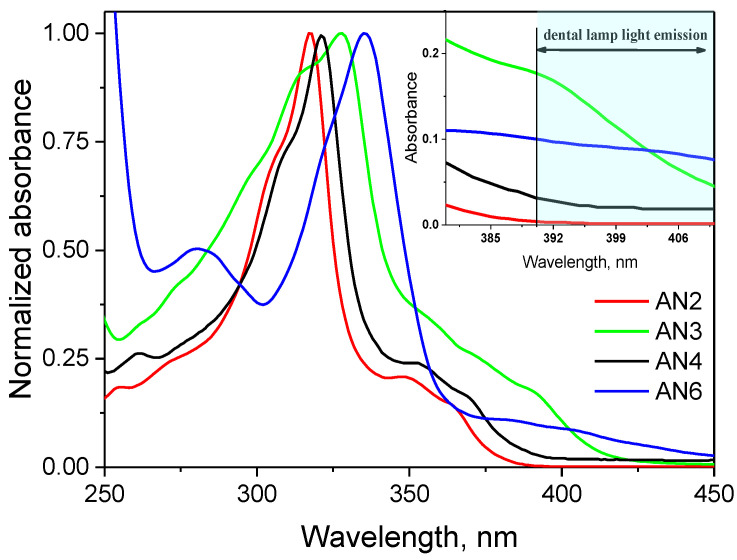
Normalized electron absorption spectra of selected dyes differing in structure, recorded in anhydrous ethanol; inset: fragments of the electronic absorption spectra of the dyes in the area of light emission of a dental lamp.

The electronic absorption spectra of the synthesized dyes were recorded in anhydrous ethanol and are shown in Figure 1. Based on them, the wavelength at the maximum absorption (*λ_max_*) and the molar absorption coefficients (*ε*) were determined (Table 1). Despite the absorption maximum is in the range of 316–363 nm, the absorption bands of the tested dyes are wide and overlap the visible area. Thanks to this, they are able to absorb the light emitted by the dental lamp. Moreover, their molar absorption coefficients at 400 nm range from about 675 dm^3^ mol^−1^ cm^−1^ to 2350 dm^3^ mol^−1^ cm^−1^ and are higher than that of camphorquinone (8 dm^3^ mol^−1^ cm^−1^ and 40 dm^3^ mol^−1^ cm^−1^ at 400 nm and 472 nm in ethyl acetate, respectively), which is the standard photoinitiator in dentistry. The molar absorption coefficients of the acenaphthoquinoxaline (AN1–AN8) derivatives range from 27,200 to 189,900 dm^3^ mol^−1^ cm^−1^ depending on their structure.

The data collected in Table 1 and shown in Figure 1 clearly indicate that the absorption band position of the dyes depends on the number of conjugated double bonds (AN1, AN2, AN6), the presence of electron donating (–OCH_3_) and electron withdrawing (–COOH) substituents (AN2, AN3, AN4), the arrangement of aromatic rings (linear or angular) (AN2, AN5, AN7), as well as the number and position of nitrogen atoms (AN2, AN6, AN8). It was observed that the modification of acenaphthoquinoxaline skeleton in all cases changed the shape of the absorption band and shifted its maximum towards longer wavelengths by about 1–47 nm (bathochromic effect) compared to the AN1 compound. The observed effect is in agreement with the electron theory of the color of dyes [61,62]. According to this theory, organic compounds having single bonds, regardless of their number, absorb light in the ultraviolet region (AN1). With the growth of closed, conjugated double bonds, the π-electron excitation energy decreases, and thus the absorption shifts to the longer wavelength region of the spectrum, which leads to the appearance of color. The introduction of a heteroatom into the conjugated aromatic system does not have a significant effect on the absorption of light, e.g., the π→π* absorption bands of AN2 and AN5 do not differ in their position. There are, however, clear differences in the intensity of the bands. The introduction of an electron donating substituent, e.g., a methoxy group, in any position to aromatic molecules always causes a bathochromic effect (AN3 vs. AN2), because this substituent is capable of donating non-binding electrons to the conjugated system (partial charge transfer from the oxygen atom to the benzene ring takes place). The red shift of the absorption band is also caused by electron acceptor substituents introduced into the conjugated molecules, e.g., a carboxyl group due to the delocalization of the π electrons even in the ground state (AN4 vs. AN2). However, the effect is not significant for the tested dyes.

It is well known that a pre-requisite for photoluminescence is the ability of a molecule to absorb light radiation leading to electronic excitation. A molecule with an enlarged stiffen structure besides fluorescence can store the absorbed energy for some time and release light later as phosphorescence [63]. Emission spectra provide valuable information about the excited states of a molecule and about the mechanism of energy transfer between molecules or different states of the same molecule [64]. Both fluorescence and phosphorescence were observed for the tested dyes.

**Figure 2:**
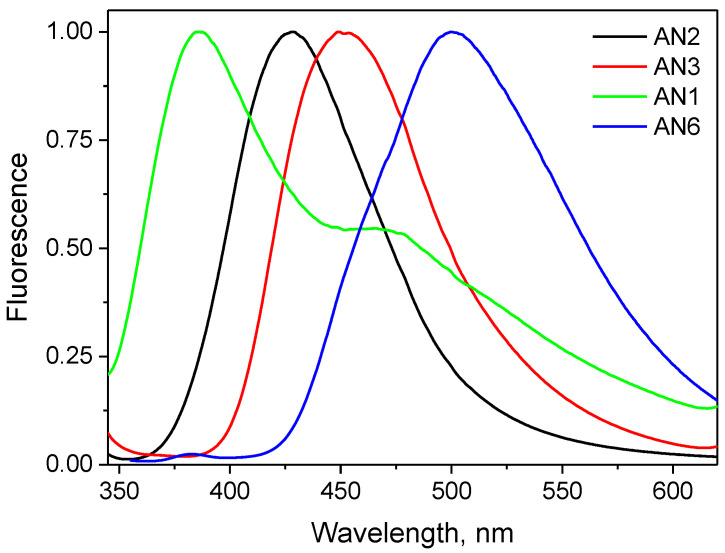
Normalized fluorescence spectra of selected compounds recorded in anhydrous ethanol at RT showing the influence of the dye structure on the position of the fluorescence band; Ex = 325 nm.

Figure 2 shows the fluorescence spectra of the selected dyes recorded in anhydrous ethanol at room temperature (RT).

As in the case of electronic absorption spectra, the position of the fluorescence maximum of the acenaphthoquinoxaline derivatives depends on their structure. The data presented in Figure 2 illustrate the bathochromic effect observed as a result of the dye structure modification. The compound AN1 has double-like fluorescence with strong peak at ca. 385 nm. The fluorescence maximum of AN2 and AN3 is at 428 nm and 449 nm, respectively, showing a gradual red shift with an increase in the number of conjugated double bonds (AN1 vs. AN2) and the introduction of electron donating (–OCH_3_) substituents (AN2 vs. AN3) and reaches 530 nm for dye AN6 (Table 1). The fluorescence maximum of the compound AN7 with a larger system of conjugated double bonds, i.e., two additional aromatic rings, is at 445 nm. So, the dye reveals hypsochromic shift in fluorescence band position compared to AN6 and bathochromic shift with respect to AN2. This means that the additional nitrogen atoms in the dye molecule is responsible for shifting the fluorescence spectrum to the shorter wavelength (higher frequency) region. A similar blue shift is observed for compound AN8 with an angular arrangement of aromatic rings compare to AN6, and AN4 having an electron withdrawing (–COOH) substituent with respect to AN2. For acenaphthoquinoxaline derivatives with increasing conjugated double bonds, the fluorescence spectra become structureless and the bands broaden. 

**Figure 3:**
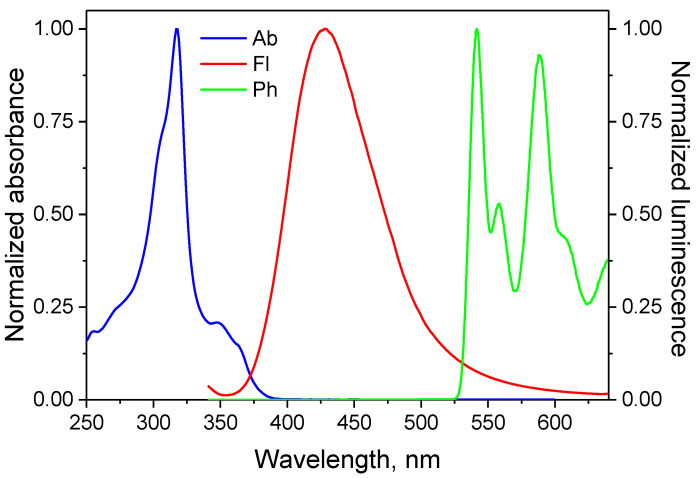
Electronic absorption (Ab), fluorescence (Fl) and phosphorescence (Ph) spectra of acenaphto[1,2-b] quinoxaline (AN2) in anhydrous ethanol; the absorption and fluorescence spectra were recorded at RT whereas the phosphorescence spectra were recorded at 77 K; Ex = 325 nm.

The large Stokes shifts (such as 10,976 cm^−1^ for AN5) and the broadening of the emission bands indicate an intramolecular charge transfer (ICT) character for the excited state [65]. A lack of symmetry between the absorption and emission spectra, as shown in Figure 3 (the remaining spectra are given in Supporting Information, Figure S1), suggests a structural relaxation of the Franck-Condon singlet excited state.

Since the triplet state energy is lower than the corresponding singlet state energy, the phosphorescence spectrum is expected to be a longer wavelength than the fluorescence spectrum [64]. The spectra presented in Figure 3 illustrate the relationships between the absorption and emission spectra for the studied dyes. The position of the phosphorescence maxima of the acenaphthoquinoxaline derivatives are summarized in Table 1.

The formation of the triplet state is a desirable feature for the application of dyes as sensitizers in visible light photoinitiating systems [21,66]. Since the energy of the visible light is not enough to homolytic cleavage of the bond in the dye molecule, radicals, active in the initiation of polymerization, are formed in bimolecular processes, i.e., the photoinduced intermolecular electron transfer (PET) process [67,68,69,70,71], in which absorbed photons initiate an electron transfer from an electron donor molecule to an electron acceptor molecule. This process is effective if both molecules are at the appropriate distance from each other. Thus, in two-component photoinitiating systems without electrostatic interactions in the ground state, diffusion and the excited state lifetime of the molecule play an important role. The main steps of the photoinitiation mechanism are the quenching of the excited singlet or triplet state of the chromophore and subsequent post-reactions that produce free radicals. Since the lifetime of the excited triplet state is much longer than that of the singlet state [63], the PET process is more efficient. As illustrated in Figure 3 and Figure S1, the synthesized acenaphthoquinoxaline derivatives form a long-lived triplet state, which makes them effective chromophores in two-component photoinitiating systems containing a suitable co-initiator (electron donor or hydrogen atom donor).

### 3.3. Kinetic Study of Multifunctional Acrylate Polymerization

#### 3.3.1. Effect of Photoinitiator Structure

**Figure 4:**
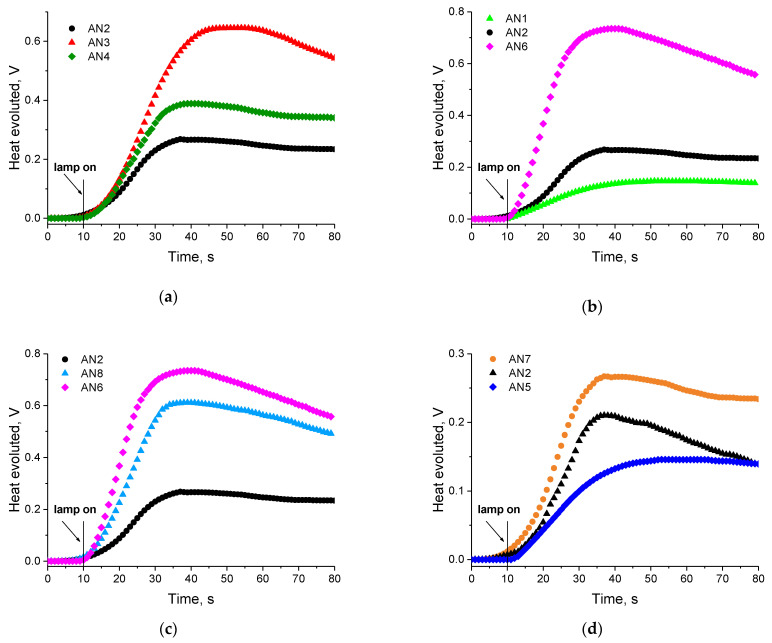
Family of kinetic curves recorded during the measurements of the heat flow emitted in the course of TMPTA polymerization photoinitiated by couples consisting of tested dyes and diMPhTAA co-initiator. The dyes used differ in: (**a**) the type of substituent; (**b**) the number of conjugated double bonds; (**c**) the arrangement of aromatic rings; (**d**) the number and position of the nitrogen atoms. The plots illustrate the effect of the sensitizer structure on the initiation efficiency of TMPTA polymerization. The co-initiator concentration was 0.1 M, optical density of the dyes was 1.8 at an excitation wavelength (400 nm), light intensity of dental lamp was 30 mW/cm^2^.

**Figure 5:**
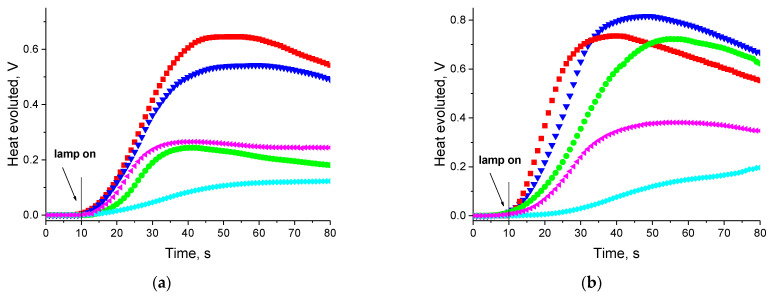
Family of kinetic curves recorded during the measurements of the heat flow emitted in the course of TMPTA polymerization photoinitiated by either (**a**) AN3 or (**b**) AN6 paired with different co-initiators (0.1 M); ■—diMPhTAA, ▼—PhIdiAA, ●—PhTAA, ◀—MPhAA, ♦—PhAA. The plots illustrate the effect of the electron donor on the initiation efficiency of TMPTA polymerization. The co-initiator concentration was 0.1 M, optical density of the dyes was 1.8 at an excitation wavelength (400 nm), light intensity of dental lamp was 30 mW/cm^2^.

Figure 4 and Figure 5 show the TMPTA radical polymerization curves initiated by synthesized dyes in the presence of diMPhTAA as an electron donor. The analysis of the curves and the rates of photoinitiated polymerization, *R_p_* collected in Table 2 shows that the efficiency of TMPTA photoinitiation significantly depends on the structure of the acenaphthoquinoxaline derivatives.

As shown above, the presence of an electron donating (–OCH_3_) or an electron withdrawing (–COOH) substituents, the increase in the number of conjugated double bonds, the arrangement of aromatic rings, and the number and location of nitrogen atoms in dye molecule causes a significant variation in the photoinitiating abilities of the two-component systems. The acenaphthoquinoxaline derivative with an electron donating substituent (AN3) in the quinoxaline ring reveals better photoinitiating abilities than AN2. Also, an increase in the number of double bonds due to the presence of an additional aromatic ring (AN6), increases the rate of TMPTA polymerization. In turn, acenaphthoquinoxaline derivatives containing three and four nitrogen atoms in the molecule (AN5 and AN7) sensitize the initiation of the chain reaction ineffectively.

**Figure 6:**
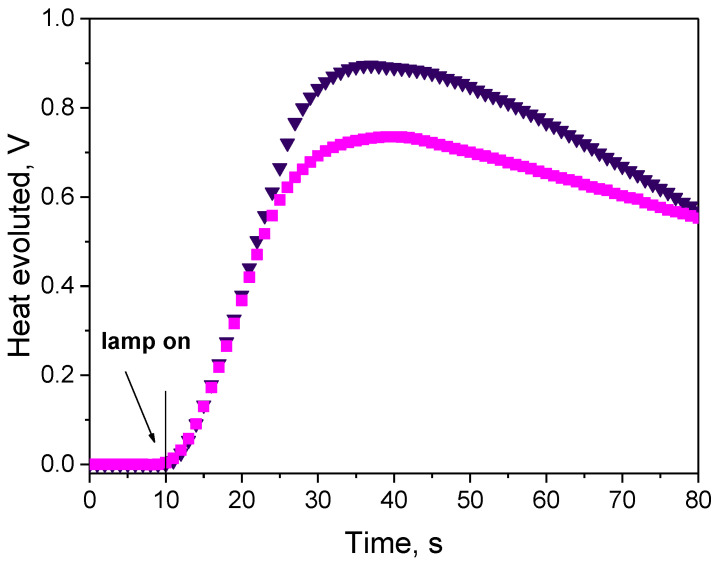
Comparison of the photoinitiation ability of the acenaphto[1,2-b]benzo[g]quinoxaline (AN6) to the camphorquinone (CQ). The tested photoinitiating systems possess the same co-initiator, (3,4-dimethoxyphenylthio)acetic acid (diMPhTAA), and different light absorber (sensitizer). The kinetic curves were recorded during the measurements of the heat flow emitted in the course of TMPTA polymerization photoinitiated by ■—AN6–diMPhTAA and ▼—CQ–diMPhTAA couples. The co-initiator concentration was 0.1 M, optical density of the sensitizers was 1.8 at an excitation wavelength (400 nm), light intensity of dental lamp was 30 mW/cm^2^.

A crucial effect on the rates of TMPTA radical polymerization also has the type of co-initiator and its property. This is fully confirmed by the data shown in Figure 6 for two sensitizers AN3 and AN6. The choice of electron donors was based on our previous research on the photoinitiated polymerization process [19,23,29,72,73]. In order to evaluate the effect of the different co-initiators on the photoinitiating abilities of the tested photoredox pairs, PhAA was used as a benchmark electron donor. Analysis of the results shown that the presence of an oxygen atom in the co-initiator structure (PhAA) reduces the value of the photopolymerization rate compared to the electron donors containing the sulfur atom (PhTAA) and the nitrogen atom (PhIdiAA). This is connected with a more efficient PET process due to different reactivity of the radicals obtained during secondary reactions that follow the electron transfer process. The following radicals are formed from the co-initiators: $C_{6}H_{5}O\dot{C}HCOOH$ and $C_{6}H_{5}O\dot{C}H_{2}$ from phenoxyacetic acid, $C_{6}H_{5}S\dot{C}HCOOH$ and $C_{6}H_{5}S\dot{C}H_{2}$ from thiophenoxyacetic acid and $C_{6}H_{5}NH\dot{C}{\left(COOH\right)}_{2}$ and $C_{6}H_{5}NH\dot{C}H_{2}$ from N-phenyiminodiacetic acid. This is confirmed by the analysis of products formed from the electron donors under photoreduction conditions. For example, the thiophenoxyacetic acid used as an electron donor gives thioanisole, thiophenol, CO_2_ and diphenyl disulfide [74]. Our studies [75] based on the ^1^H-NMR technique with the use of standards showed that the post-reaction mixture also contained bis(phenylthioethane) and bis(phenylthiomethane). 

In the case of PhIdiAA, the heteroatom the presence of second carboxylic group is responsible for the increase of the rate of TMPTA polymerization. According to the literature data [76], it is known that initiation mechanism based on carboxylic acids obeys proton transfer with the possibility of a decarboxylation process to form radicals, which initiate polymerization (Scheme 2). 

The results also revealed that the co-initiators containing a methoxy group (MPhAA, MPhTAA) show good photoinitiating ability. The initial rates of photoinitiated polymerization containing diMPhTAA as an electron donor paired with AN1–AN8 dyes were approximately 10 times faster than the polymerization rates obtained in the presence of PhAA and ca. twice in respect to MPhTAA. The methoxy group in the electron donor molecule favors the increase of the photopolymerization rate probably because they have lower oxidation potential (1.170 V—MPhTAA and 0.779 V—diMPhTAA) than PhAA (1.450 V) (Table 3), which facilitates the electron donation in the PET process.

To verify the photoinitiating ability of the tested systems, the initial rates of radical polymerization photoinitiated by acenaphthoquinoxaline derivative (AN1–AN8)—carboxylic acid couples were comparable to the rate obtained for a polymerization initiated by CQ commercial photoinitiator used in dentistry (Figure 6). To keep the same experimental conditions (number of absorbed photons), the concentration of CQ in the composition was 160 times higher than the tested sensitizers due to lower molar absorption coefficient in the visible range (8 dm^3^ mol^−1^ cm^−1^ vs. 1300 dm^3^ mol^−1^ cm^−1^ at 400 nm). This made it possible to obtain thick polymer layers (3 mm) that did not contain significant amounts (approx. 0.675 M) of unreacted CQ and products resulting from its decomposition during the photochemical reaction. The temperature of the photopolymerization process initiated by the acenaphthoquinoxaline—acetic acid systems was lower than that of the commercial photoinitiator, which allows the safe use of these systems in dentistry. In addition, the initial rates of TMPTA polymerization are similar and the hard polymer glaze is obtained after ca. 30 seconds of irradiation (Figure 6). 

#### 3.3.2. Influence of Thermodynamics Parameter

The radical polymerization initiated by the intermolecular electron transfer mechanism proceeds through many stages, the main of which is electron transfer from the electron donor molecule to the electron acceptor molecule in the singlet (or triplet) excited state. The photoinduced electron transfer process is limited, inter alia, by thermodynamic factors, i.e., the thermodynamic potential of an activation of the electron transfer process, Δ*G_el_*. The value of Δ*G_el_* can be determined experimentally using the Rehm–Weller Equation (1): [69,77]

(1) $$\Delta G_{el}=E_{ox}-E_{red}-\frac{Ze^{2}}{\varepsilon a}-E_{T}^{00}$$

where: *E_ox_* is the oxidation potential of the co-initiator, *E_red_* is the reduction potential of the dyes, $E_{T}^{00}$ is the excited state energy (the energy of T_1_→S_0_ transition), and *Ze*^2^/*εa* is the Coulombic energy, which is considered negligible with respect to the overall magnitude of the Δ*G* in the present system. 

To calculate Δ*G_el_*, it is necessary to know the oxidation potential of the co-initiators (Table 3), the reduction potential of the electron acceptors (Table 4) and the transition energy T_1_→S_0_. The determined values of Δ*G_el_* are summarized in Table 4. The Δ*G_el_* values for the tested photoredox pairs are in the range of −0.130 eV to −0.471eV (from −12 kJ mol^−1^ to −45 kJ mol^−1^). The negative values of Δ*G_el_* indicate the photoinduced electron transfer process is thermodynamically allowed.

Equation (2) indicates that if the process of intermolecular electron transfer between the excited dye molecule and the electron donor molecule limits a polymerization rate, a parabolic relationship between the polymerization rate and the thermodynamic potential of an activation of the electron transfer process, Δ*G_el_* should be observed [68,70,78].

(2) $$\ln R_{p}=A-\frac{{\left(\lambda +\Delta G_{el}\right)}^{2}}{8\lambda RT}$$

where: *A* for the initial time of polymerization is:

(3) $$A=\ln k_{p}-0.5\ln k_{t}+1.5\ln\left[M\right]+0.5\ln I_{A}$$

where: *k_p_* and *k_t_* are the polymerization and termination rate constant, respectively, [*M*] is the monomer concentration, *I_A_* is intensity of absorbed light, *λ* is the reorganization energy necessary to reach the transition states of both excited molecule and solvent molecules.

**Figure 7:**
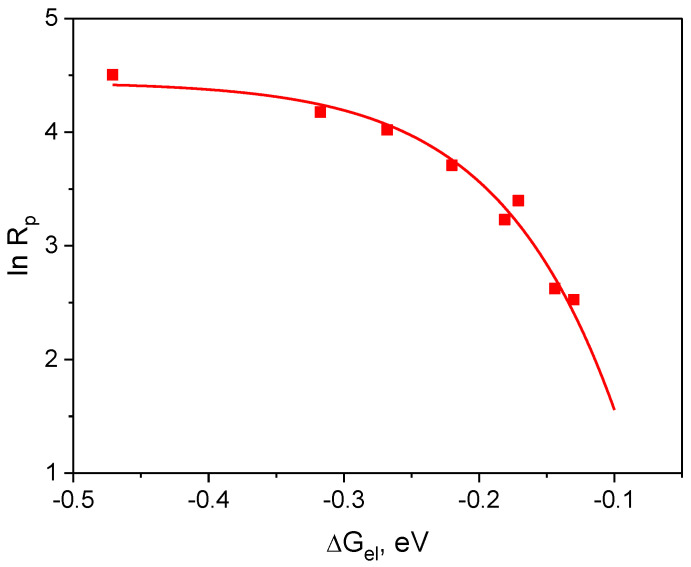
The dependence of the initial polymerization rate on the thermodynamic potential of an activation of the electron transfer process from the electron donor to the excited triplet state of acenaphthoquinoxaline dyes.

The correlation resulting from Equation (2) is shown in Figure 7.

According to the plot shown in Figure 7, the correlation between the initial polymerization rate and the thermodynamic activation potential of the electron transfer process is a fragment of the parabola, which indicates that the tested photoredox pairs behave in accordance with the classical theory of photoinduced electron transfer process [70,71]. The PET process is a polymerization rate-limiting process. Experimental points are located in the so-called normal Marcus region, i.e., the region where the rate of the process increases as its driving force increases [67,68,70,71].

#### 3.3.3. Photoinitiation by Hydrogen Atom Transfer Mechanism

In addition to the PET mechanism, other classical photochemical reactions can be used to generate radicals initiating the polymerization reaction, i.e., the abstraction of a hydrogen atom from the proton donor molecule by the dye in the excited state (Scheme 3). The resulting radicals can recombine to give various products. The rate of the photoinduced hydrogen transfer process depends on the energy needed to break the bond between hydrogen atom and the rest of the hydrogen donor molecule. Typical hydrogen donors are: alcohols, amines, thiols, phenols and others, while the most commonly used hydrogen acceptors are: benzophenone, Michler’s ketone, thioxanthone derivatives, benzil and anthraquinone [14].

**Figure 8:**
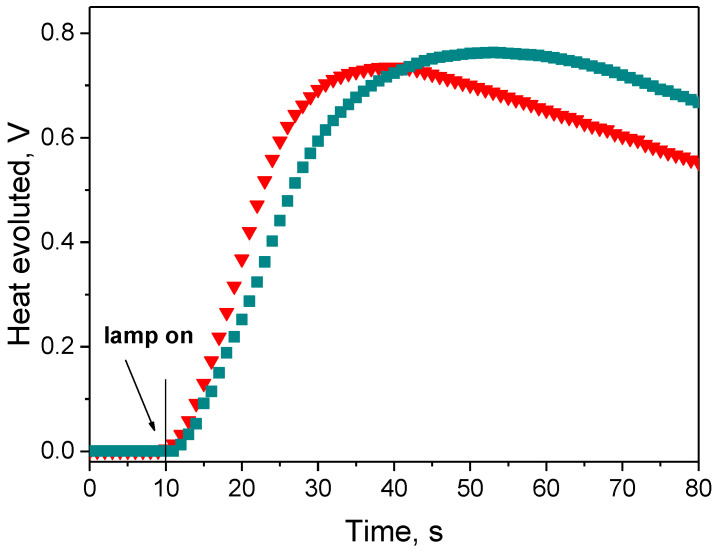
Comparison of the acrylate monomer polymerization initiated by either an electron transfer or hydrogen atom transfer mechanism. The tested photoinitiating systems possess the same light absorber, acenaphto[1,2-b]benzo[g]quinoxaline (AN6) and different co-initiator, i.e., an electron donor ▼—diMPhTAA and a hydrogen atom donor ■—MBX. The kinetic curves were recorded during the measurements of the heat flow emitted in the course of TMPTA polymerization. The co-initiator concentration was 0.1 M, optical density of the sensitizers was 1.8 at an excitation wavelength (400 nm), light intensity of dental lamp was 30 mW/cm^2^.

The 2-mercaptobenzoxazole (MBX) was used as hydrogen atom donor in our studies. Analysis of the polymerization curves shown in Figure 8 reveals that the synthesized dyes are effective sensitizers for hydrogen atom donors and the photoinitiation of the polymerization reaction can also take place through the mechanism of intermolecular hydrogen atom transfer. 

The data collected in Table 2 indicates that the photopolymerization rates obtained for the photocurable compositions containing MBX as the hydrogen atom donor are comparable with the compositions containing the diMPhTAA electron donor.

In summary, the obtained acenaphthoquinoxaline derivatives can be used as sensitizers in visible light photoinitiator systems containing as co-initiator both electron donors and hydrogen atom donors. The radicals initiating the TMPTA polymerization in the tested systems are formed by the intermolecular electron transfer mechanism, followed by a proton transfer between the components of the radical ion pair or decarboxylation of the radical cation depending on the polarity of the solvent or the abstraction of the hydrogen atom from 2-mercaptobenzoxazole in the case of hydrogen atom transfer photoinitiators.

#### 3.3.4. Dental Test Composition

**Figure 9:**
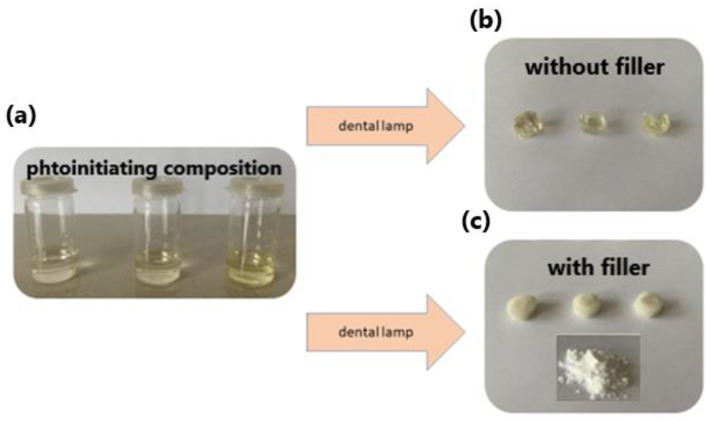
Comparison of the acrylate monomer polymerization photoinitiated by couples consisting of tested dyes and diMPhTAA co-initiator in the presence and absence of a glass ionomer, a filler for dental fillings. The co-initiator concentration was 0.1 M, optical density of the sensitizers was 1.8 at an excitation wavelength (400 nm), light intensity of dental lamp was 30 mW/cm^2^. (**a**) Photoinitiating compositions containing AN2 (left), AN3 (center) and AN8 (right) sensitizers, respectively, (**b**) glazes obtained upon irradiation of the photoinitiating compositions without a dental filler and (**c**) dental fillings resulting from polymerization of the photoinitiating compositions containing a filler.

As stated above, the tested photoinitiator systems are very promising candidates for practical application in dentistry; therefore we performed polymerization experiments in the presence of a glass ionomer, the filler for dental fillings. The glass ionomer material includes fluor-aluminum-silicon glass and organic acids. Figure 9 shows the prepared photoinitiating compositions, the glazes obtained in the photopolymerization process and the fillings resulting from the photopolymerization initiated by the tested systems.

It is clearly seen that the photoinitiating composition containing filler, i.e., the glass ionomer, upon dental lamp irradiation, gave the natural color dental fillings (Figure 9c). What is more, low concentration of the sensitizers in the photopolymerizing composition ca. 10^−4^ mol dm^−3^ allows for obtaining thick polymer layers of about 3 mm. This suggests that the two-component photoinitiating systems based on the synthesized dyes and acetic acids tested may have potential applications in dentistry.

## 4. Conclusions

Modification of acenaphthoquinoxaline skeleton resulted in dyes that differed in the type of substituents, the number of conjugated double bonds, the arrangement of aromatic rings, as well as the number and location of nitrogen atoms in the molecule. The modification of the structure influences the spectral properties of the tested compounds. The acenaphto[1′,2′:5,6]pyrazine[2,3-b]phenazine (AN7) reveals the most significant redshift of the absorption band with maximum at 347 nm and 363 nm, caused by the extended system of the conjugated double bonds in the linear arrengement of aromatic rings. This means that the absorption maxima of the synthesized compounds are located in the near ultraviolet region. However, since their absorption bands overlap the visible region, they have been tested as photoinitiators of acrylate polymerization operating upon the dental lamp irradiation. 

The synthesized dyes in the photo-curable composition act as photosensitizers, which paired with organic acids, initiate the TMPTA radical polymerization. The radicals initiating TMPTA polymerization are formed by the intermolecular electron transfer mechanism, followed by a proton abstraction and decarboxylation, or by the intermolecular proton (hydrogen atom) transfer mechanism in the case of 2-meracptobenzoxazole used as co-initiator. The acenaphto[1,2-b]benzo[g]quinoxaline (AN6) coupled with (3,4-dimethoxyphenylthio)acetic acid (diMPhTAA) shows the best photoinitiating abilities with a polymerization rate of 116.1 μmol/s. The efficiency of this photoinitiating system is comparable to the commercial photoinitiator, camphorquinone. 

Results show that the synthesized acenaphthoquinoxaline dyes are effective photosensitizers of carboxylic acids (PhAA, MPhAA, PhTAA, diMPhTAA, PhIdiAA), which can replace mutagenic aromatic amines in the photo-curable composition. The polymerization of acrylic monomers photoinitiated by the tested systems in the presence of glass ionomer, a filler in dental fillings, gives permanent fillings with a natural white color. This suggests that the two-component photoinitiating systems tested may have potential applications in dentistry.

## Figures and Tables

**Table 1:** Basic spectroscopic properties for the dyes tested in ethanol.

Compound	$\lambda {}_{max}^{Ab},\;\mathrm{nm}$	*ε_max_*, dm^3^ mol^−1^ cm^−^^1^	$\lambda {}_{max}^{Fl},\;\mathrm{nm}$	$\lambda_{max}^{Ph},\;\mathrm{nm}$	Δ*ν^St^*, cm^−1^
AN1	316	27,200	385.4	530.6	5699
				547.2	
				574.2	
				621.4	
AN2	317	65,200	427.6	541.4	5432
	347	14,600		587.8	
AN3	327	82,400	449	544.8	8309
				592.2	
AN4	321	92,000	395.6	542.4	5875
				589.4	
AN5	319	110,100	362.6	539.6	3769
				586.0	
AN6	335	145,800	529.8	543.4	10976
				586.8	
AN7	347	170,000	452.8	546.8	5076
	363	189,900		585.2	
AN8	339	146,200	473.4	522.4	8375
				561.4	
CQ ^1^	472	40	-	-	-

^1^ measured in ethyl acetate.

**Table 2:** Initial rate of photoinitiated polymerization (*R_p_*) for the tested photoredox pairs.

Dye	*R_p_*, μmol/s					
	PhAA	MPhAA	PhTAA	diMPhTAA	PhIdiAA	MBX
AN1	1.4	9.2	6.5	14.0	12.5	12.8
AN2	3.9	22.6	17.9	35.9	29.9	33.1
AN3	9.0	44.4	39.7	68.1	55.7	57.1
AN4	5.3	42.5	21.9	52.3	40.8	46.1
AN5	3.2	21.3	13.2	29.2	25.3	28.7
AN6	10.2	80.2	61.5	116.1	90.4	91.2
AN7	1.3	10.0	7.2	14.0	13.8	13.1
AN8	7.3	47.1	38.1	72.8	65.1	65.4
CQ	15.8	82.3	71.4	129.7	107.8	93.6

**Table 3:** The oxidation potentials of the electron donors used.

Electron Donor	*E_ox_* [V]
PhAA	1.450
MPhAA	1.170
PhTAA	1.076
diMPhTAA	0.779
PhIdiAA	0.718

**Table 4:** The reduction potential (*E_red_*) of the dyes, transition energy T_1_→S_0_ ($E_{T}^{00}$) and the thermodynamic potential of an activation of the electron transfer process (Δ*G_el_*).

Dye	*E_red_*, V	$E_{T}^{00},\;eV$	^1^ Δ*G_el_*, eV
AN1	−1.482	2.33	−0.130
AN2	−1.401	2.29	−0.171
AN3	−1.284	2.27	−0.268
AN4	−1.422	2.36	−0.220
AN5	−1.401	2.30	−0.181
AN6	−1.091	2.28	−0.471
AN7	−1.398	2.26	−0.144
AN8	−1.335	2.37	−0.317

^1^ Δ*G_el_* calculated for PhIdiAA (*E_o_*_x_ = 0.718 V) as the co-initiator.

## Data Availability

The data is not publicly available apart from the data contained in the article or Supplementary Materials.

## Supplementary Materials

The following are available online at https://www.mdpi.com/article/10.3390/ma14174881/s1, ^1^H and ^13^C NMR spectra of the synthesized compounds. Figure S1: Normalized electronic absorption, fluorescence and phosphorescence spectra of the dyes in anhydrous ethanol illustrating the influence of dye structure on the bands position.
