# Supplementary material for: Acenaphthoquinoxaline Derivatives as Dental Photoinitiators of Acrylates Polymerization
Source: Materials (Basel). 2021 Aug 27;14(17):4881. doi: 10.3390/ma14174881 (PMC8432685; doi:10.3390/ma14174881)
Supplement: Supplementary file 1 [file materials-14-04881-s001.zip › materials-1339327-supplementary.pdf]

Electronic Supplementary Information

Dye photoinitiators of radical polymerization of acrylates based on acenaphthoquinoxaline skeleton for potential application in dentistry

Ilona Pyszka [1,*], Beata Jędrzejewska [1,*]

[1]UTP University of Science and Technology, Faculty of Chemical Technology and Engineering

Seminaryjna 3, 85-326 Bydgoszcz, Poland

**Corresponding authors:*

I. Pyszka: e-mail address: Ilona.Pyszka@utp.edu.pl; Tel.: +48-52-374-9039 (IP)
B. Jędrzejewska: e-mail address: beata@utp.edu.pl; Tel.: +48-52-374-9046 (BJ)

Table of contents page

^{1}H and ^{13}C NMR spectra	2
Absorption and emission spectra in ethanol – Figure S1	16

^{1}H spectrum of **AN1**

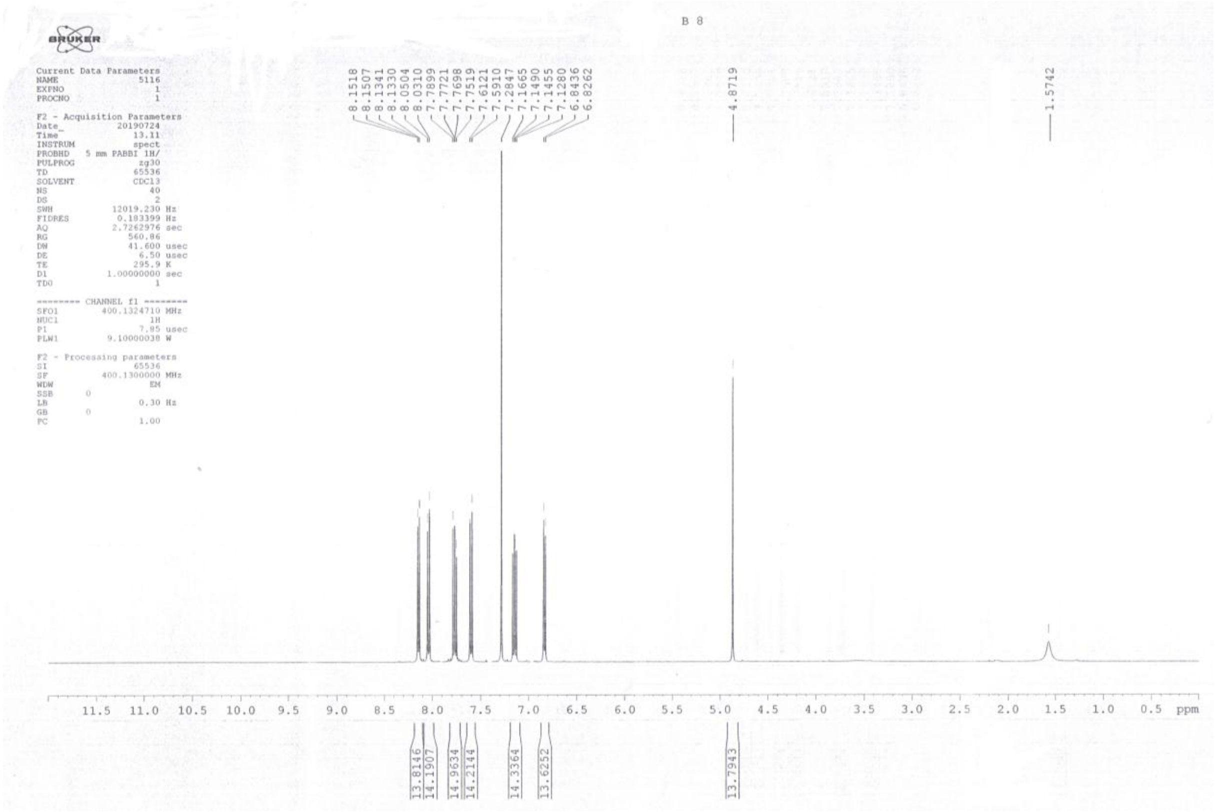

Enlarged spectrum in the range of 6-9.

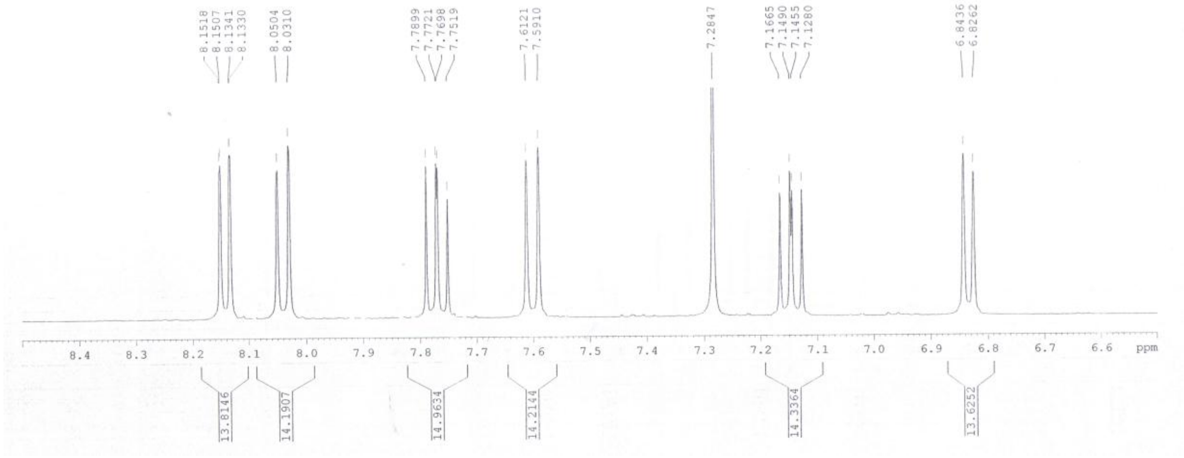

^{13}C spectrum of **AN1**

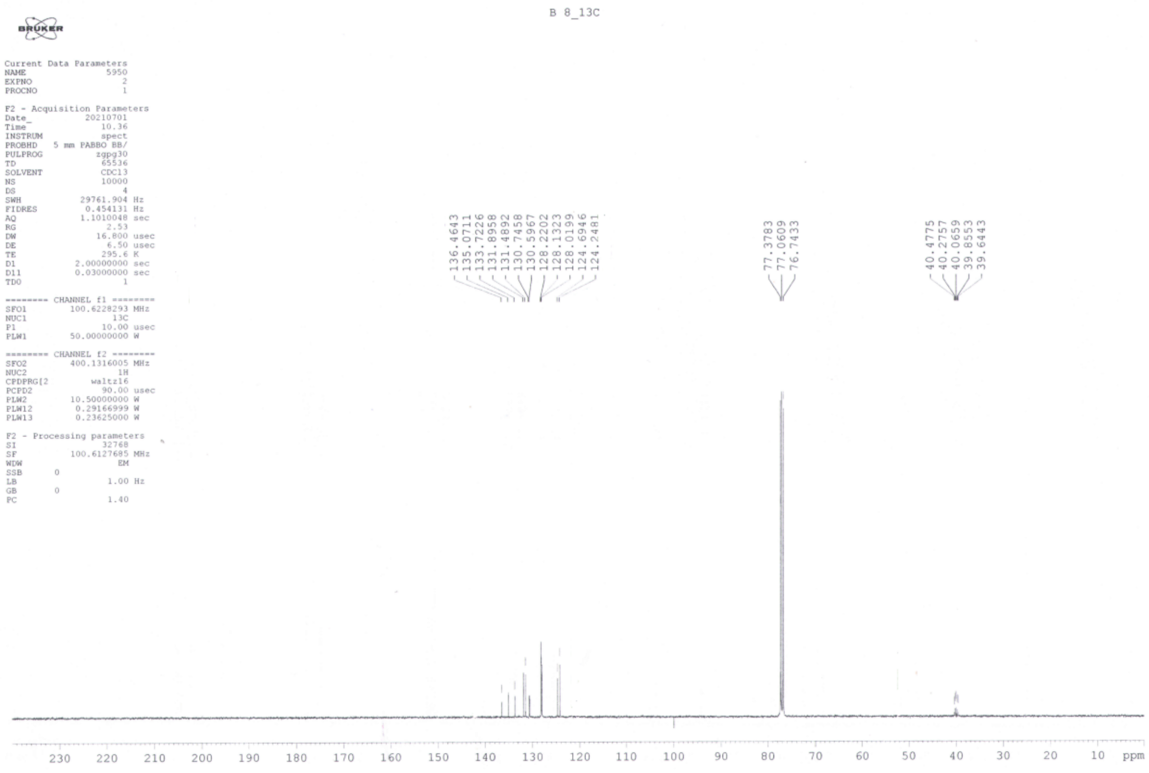

Enlarged spectrum in the range of 120-140 ppm.

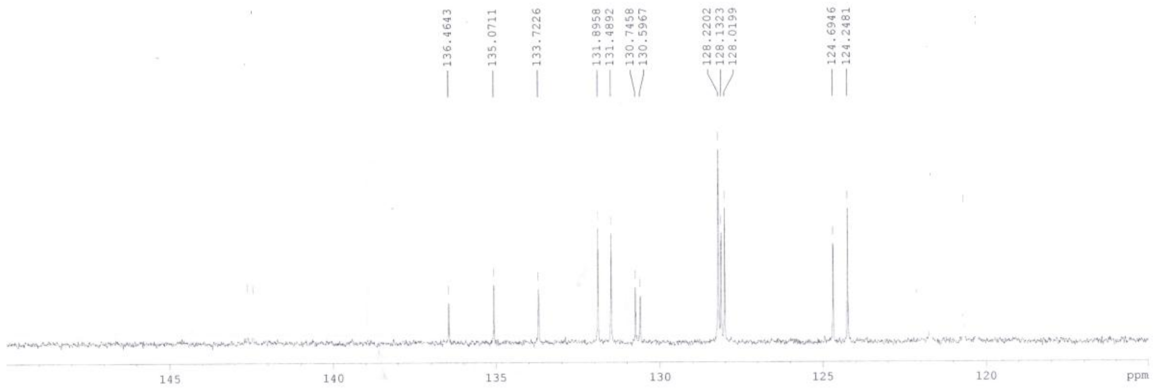

^{1}H spectrum of **AN2**

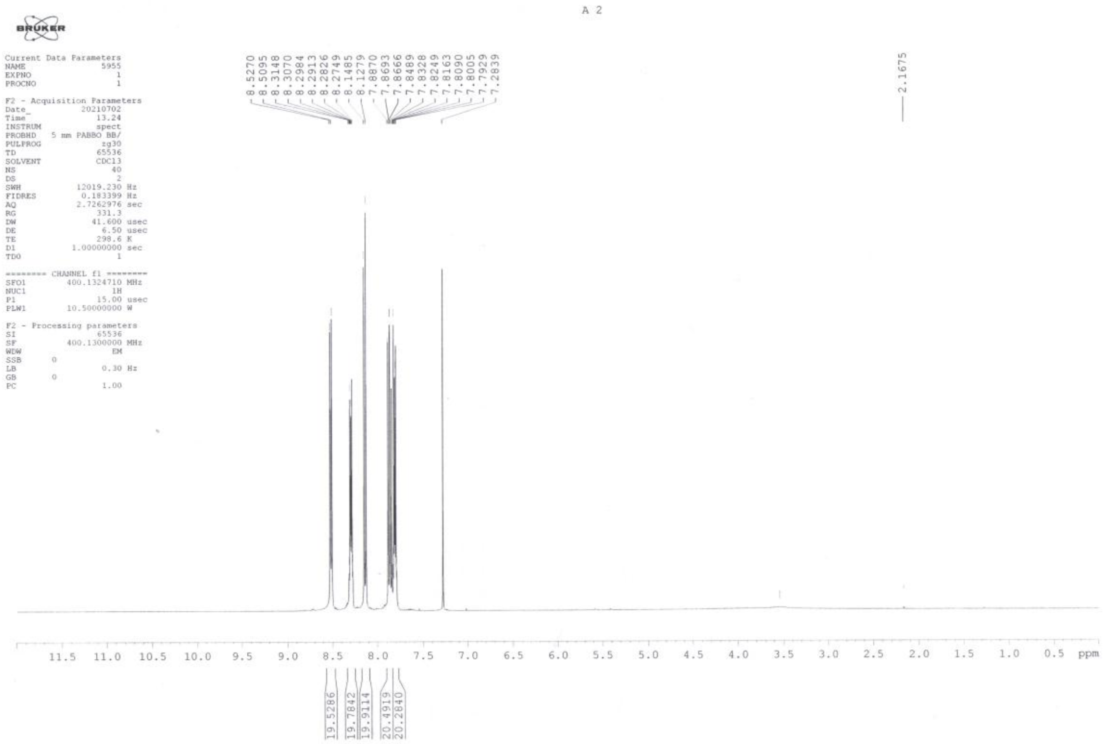

Enlarged spectrum in the range of 7-9 ppm.

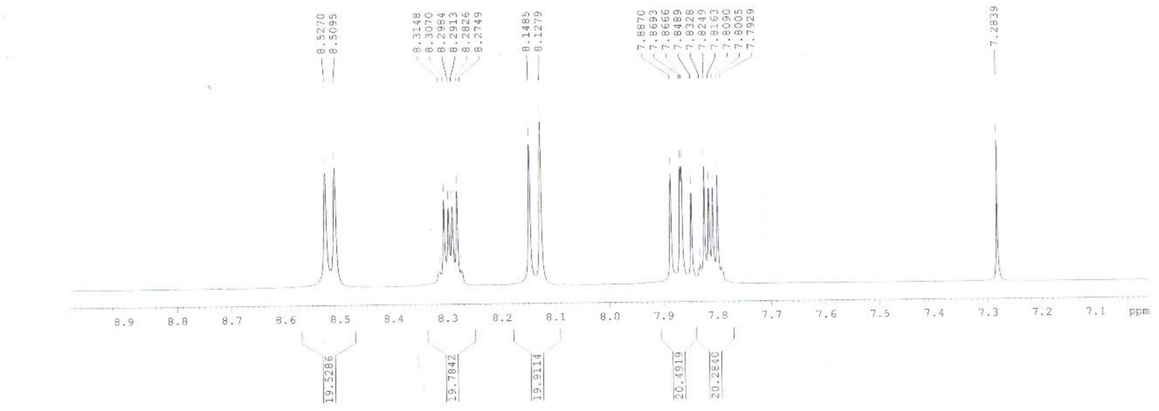

^{13}C spectrum of **AN2**

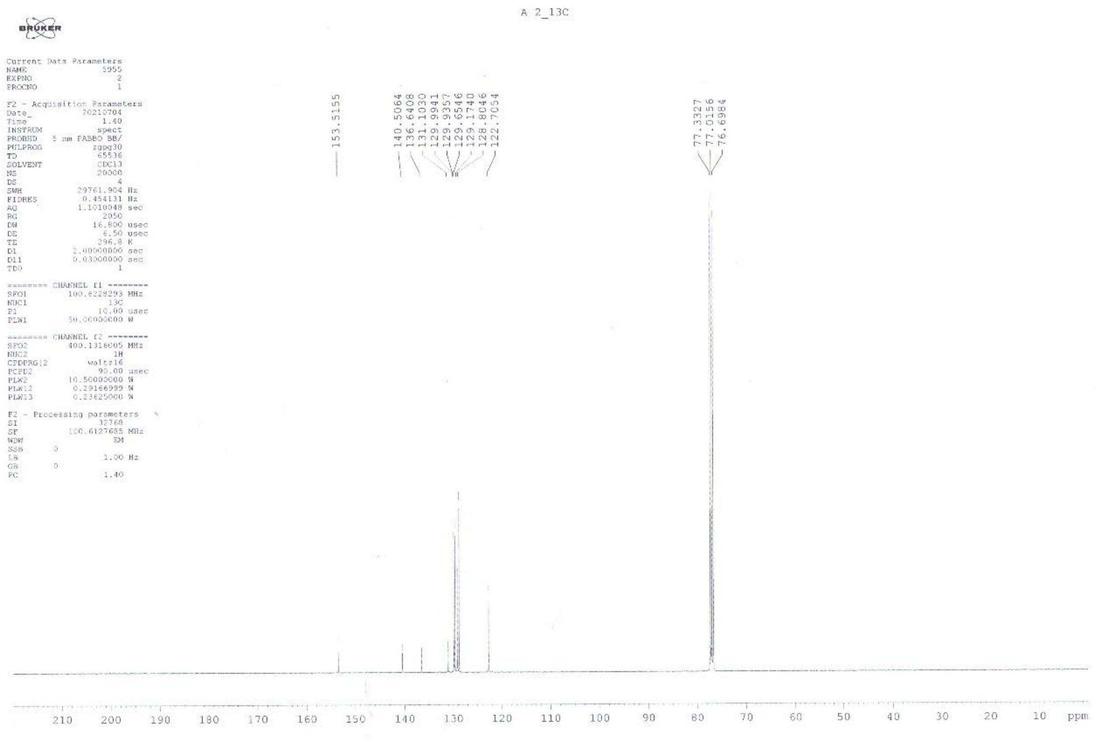

Enlarged spectrum in the range of 120-160 ppm.

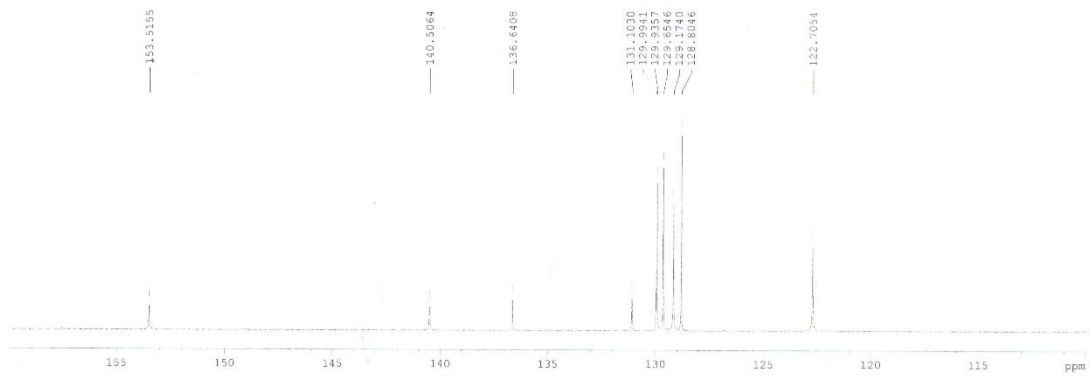

^{1}H spectrum of **AN3**

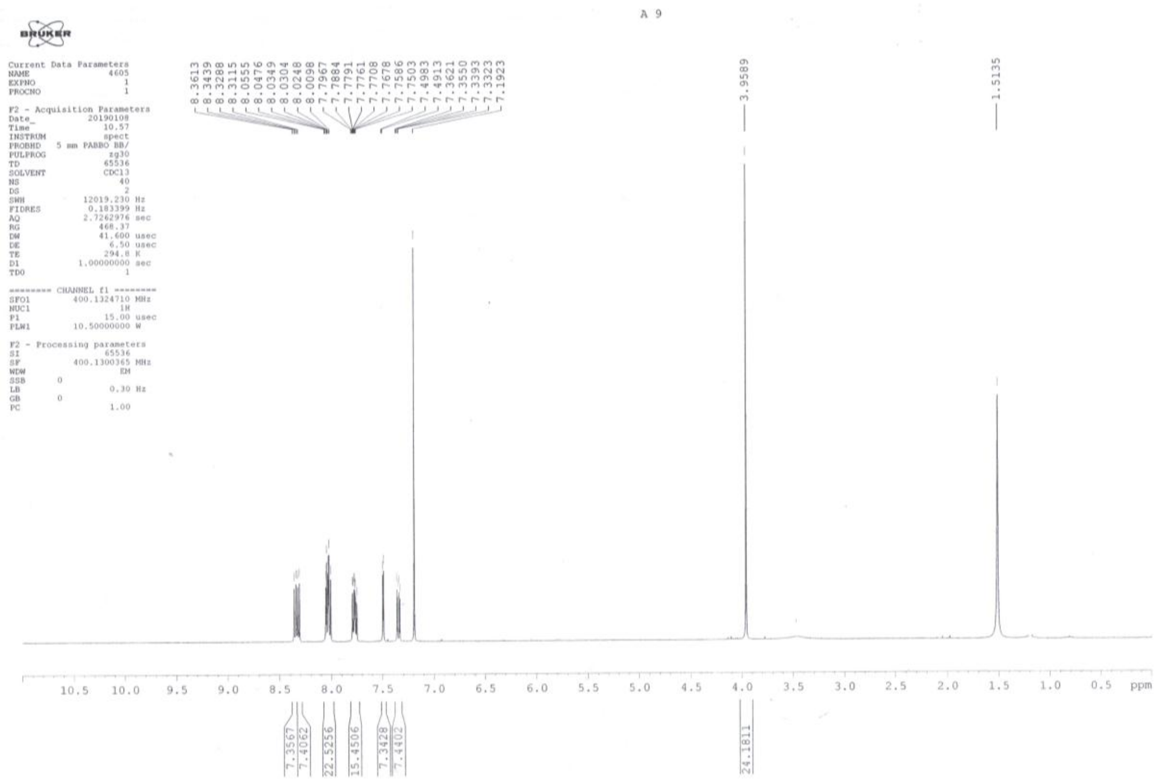

Enlarged spectrum in the range of 7-9 ppm.

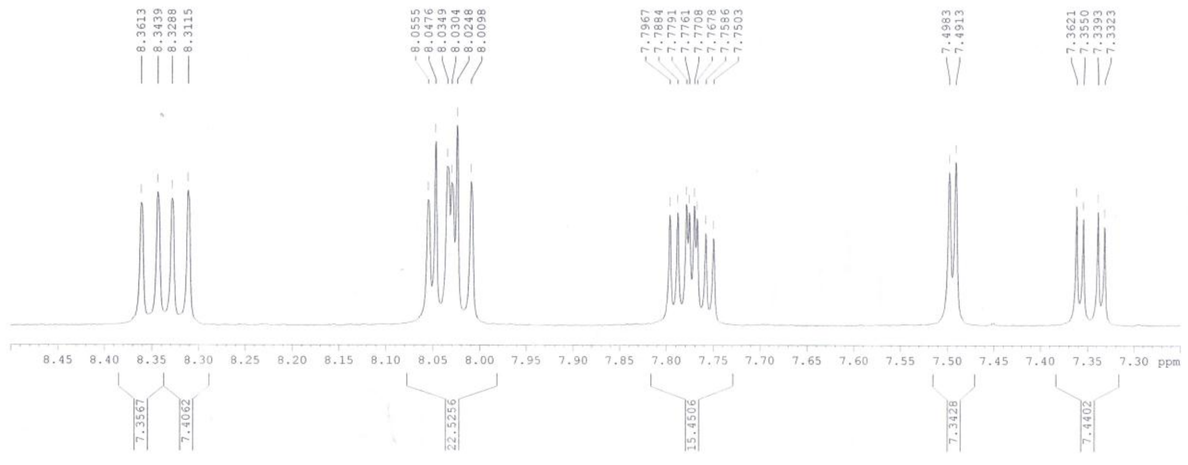

^{13}C spectrum of **AN3**

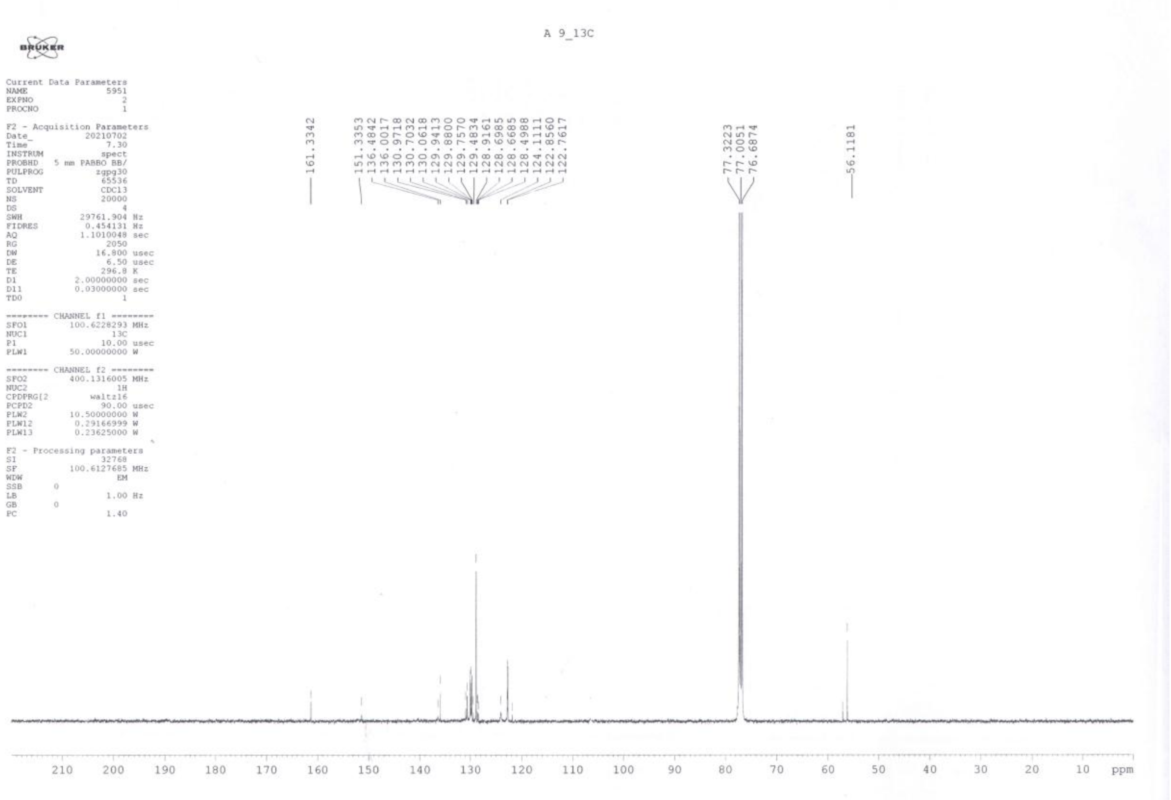

Enlarged spectrum in the range of 120-170 ppm.

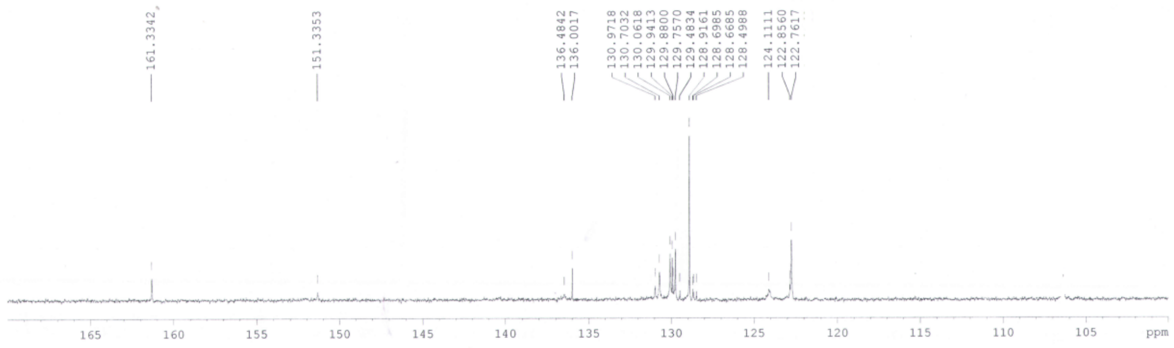

^{1}H spectrum of **AN4**

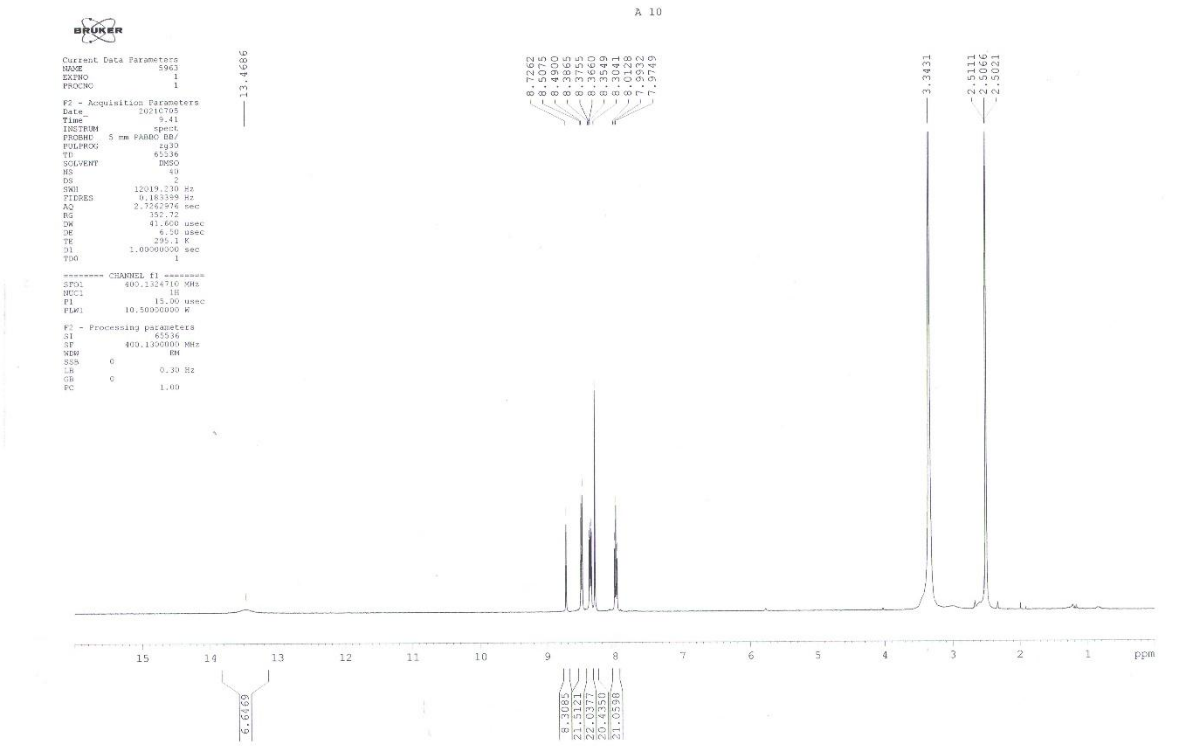

Enlarged spectrum in the range of 7-9 ppm.

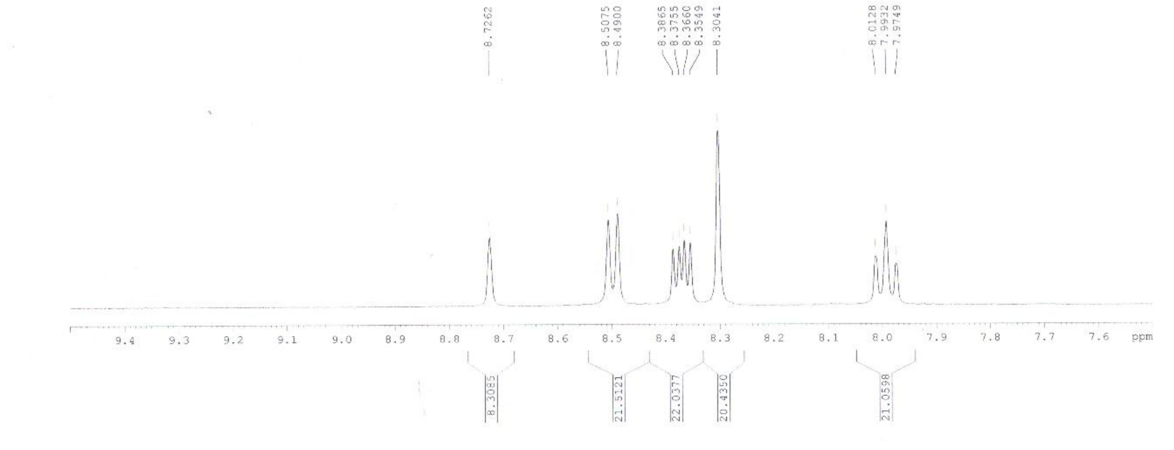

^{13}C spectrum of **AN4**

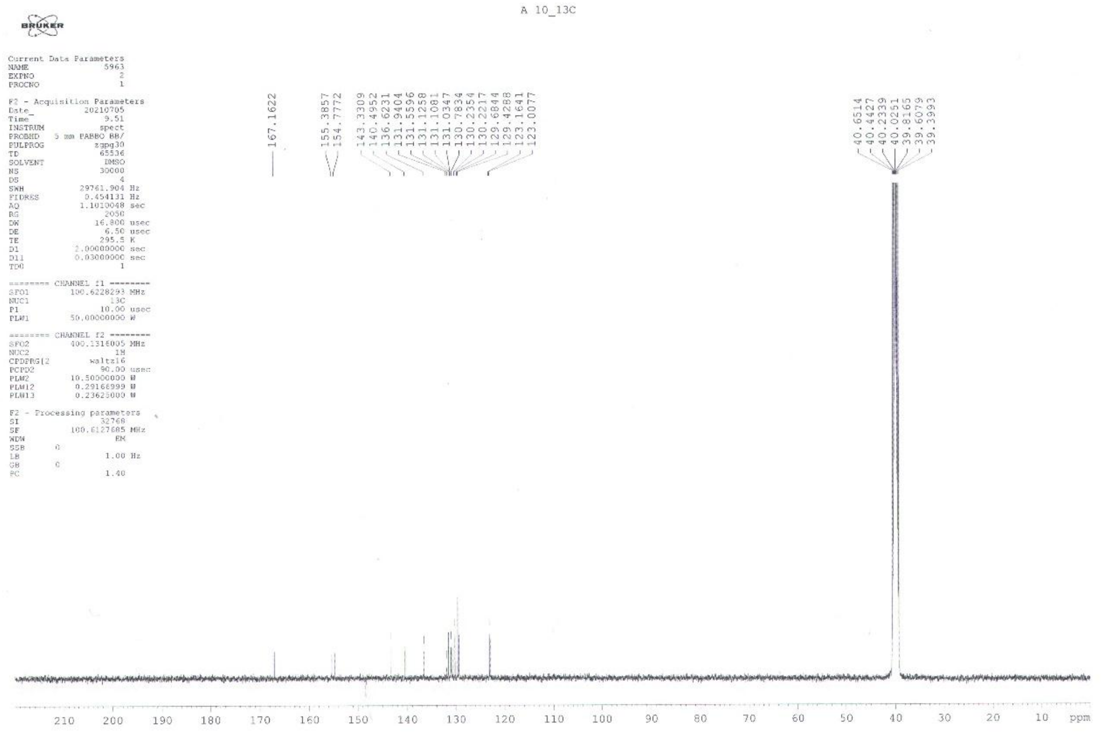

Enlarged spectrum in the range of 120-160 ppm.

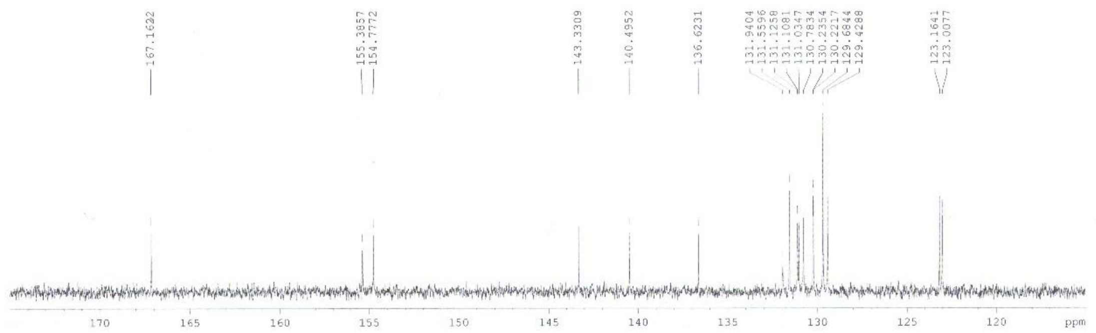

^{1}H spectrum of **AN5**

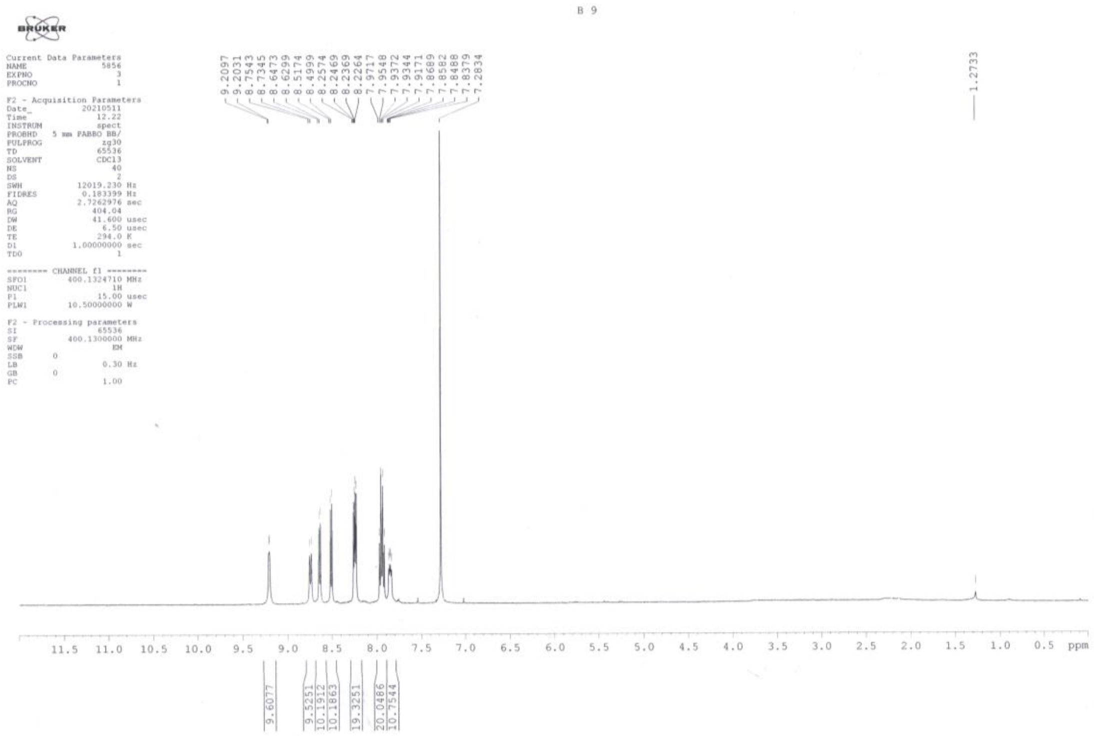

Enlarged spectrum in the range of 7-10 ppm.

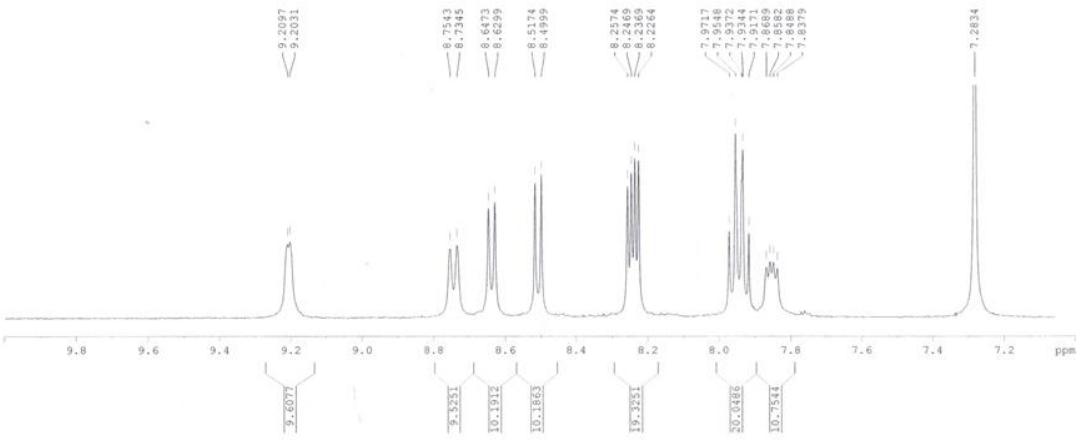

^{13}C spectrum of **AN5**

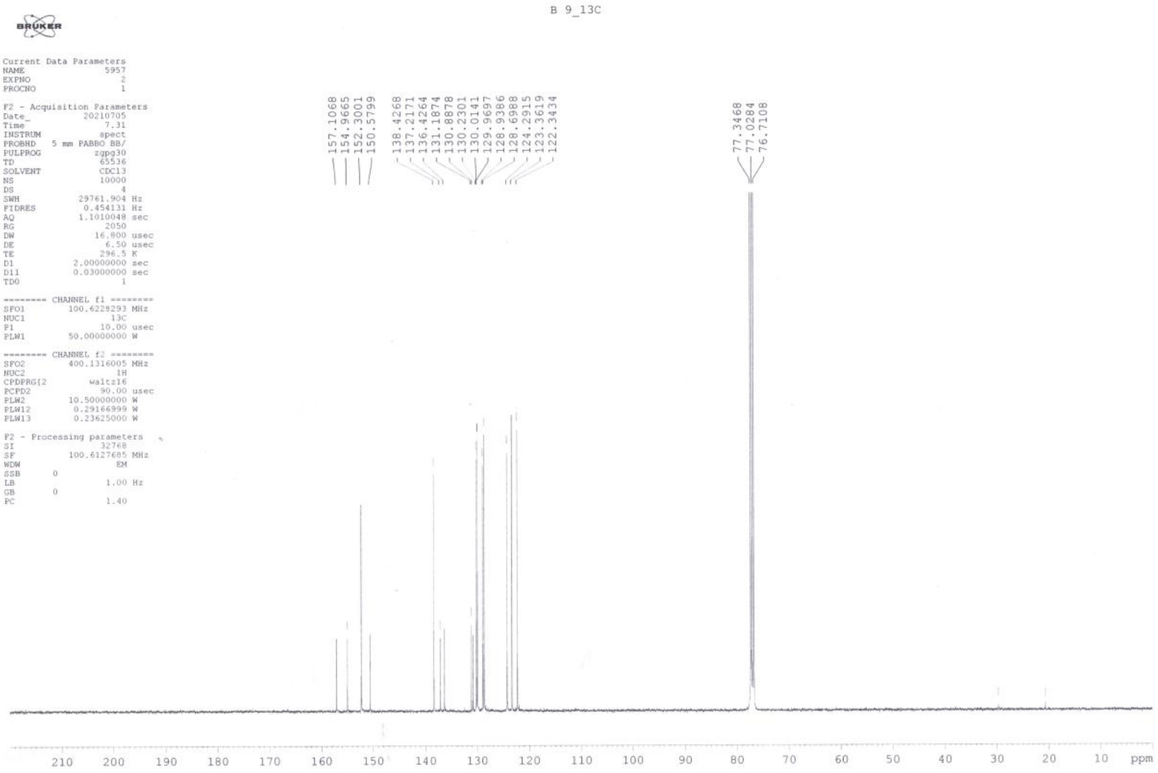

Enlarged spectrum in the range of 120-160 ppm.

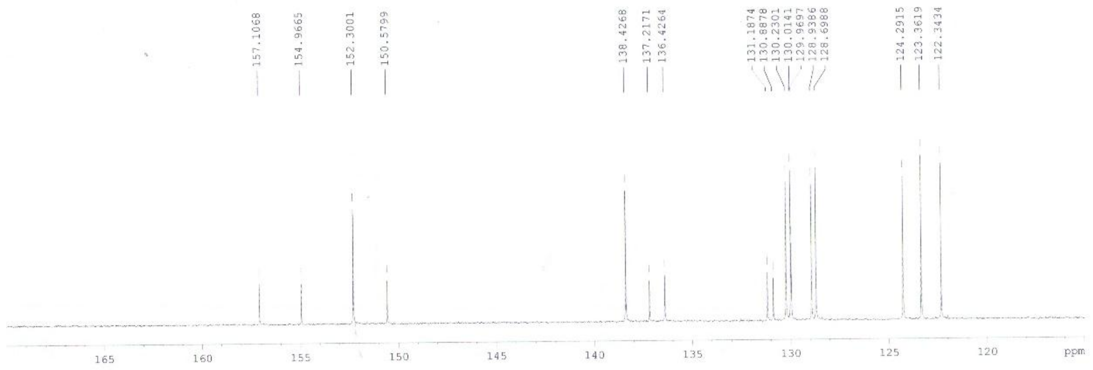

^{1}H spectrum of **AN6**

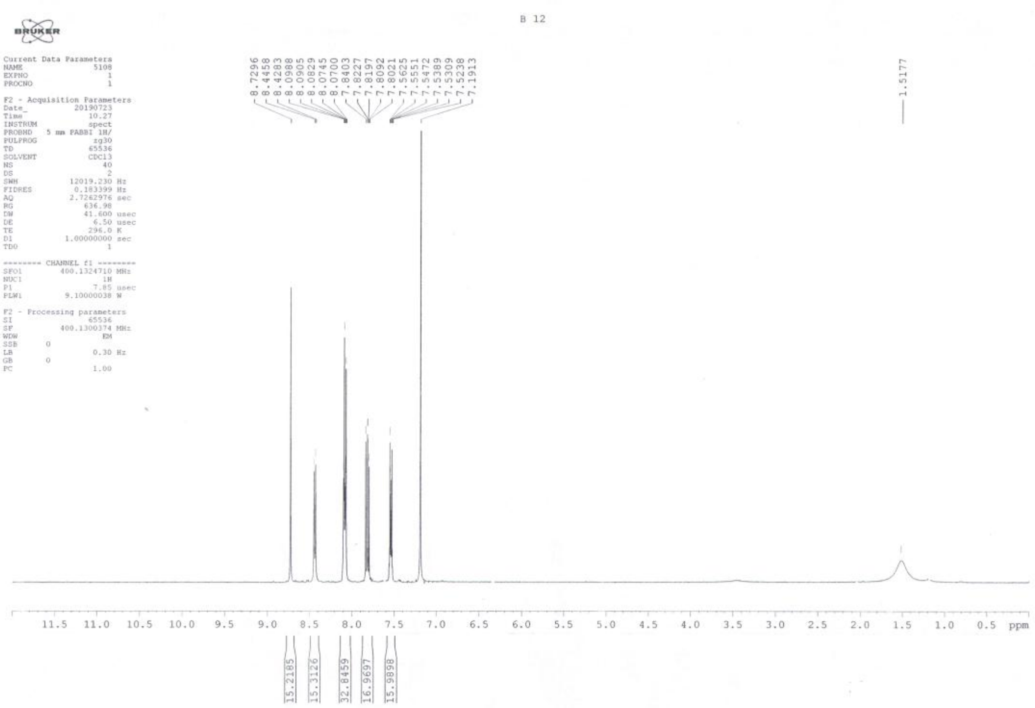

Enlarged spectrum in the range of 7-9 ppm.

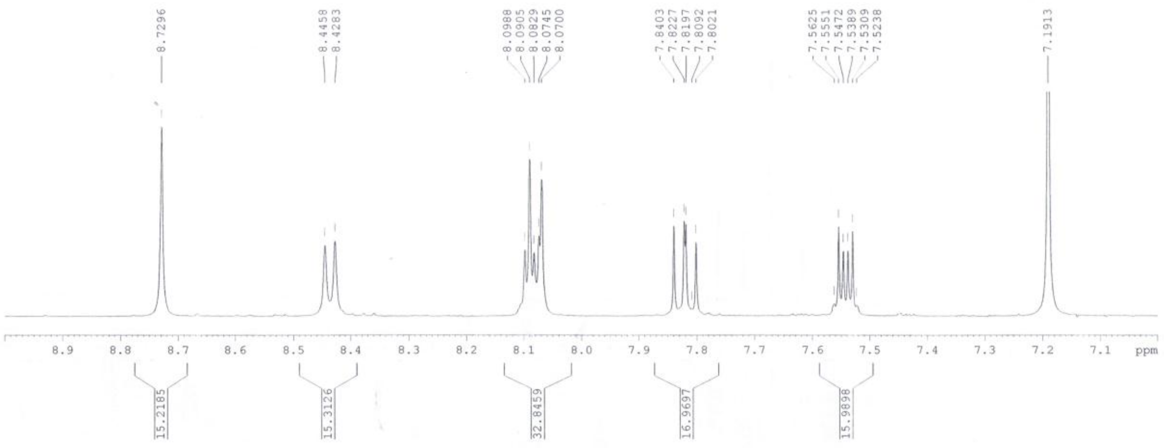

^{13}C spectrum of **AN6**

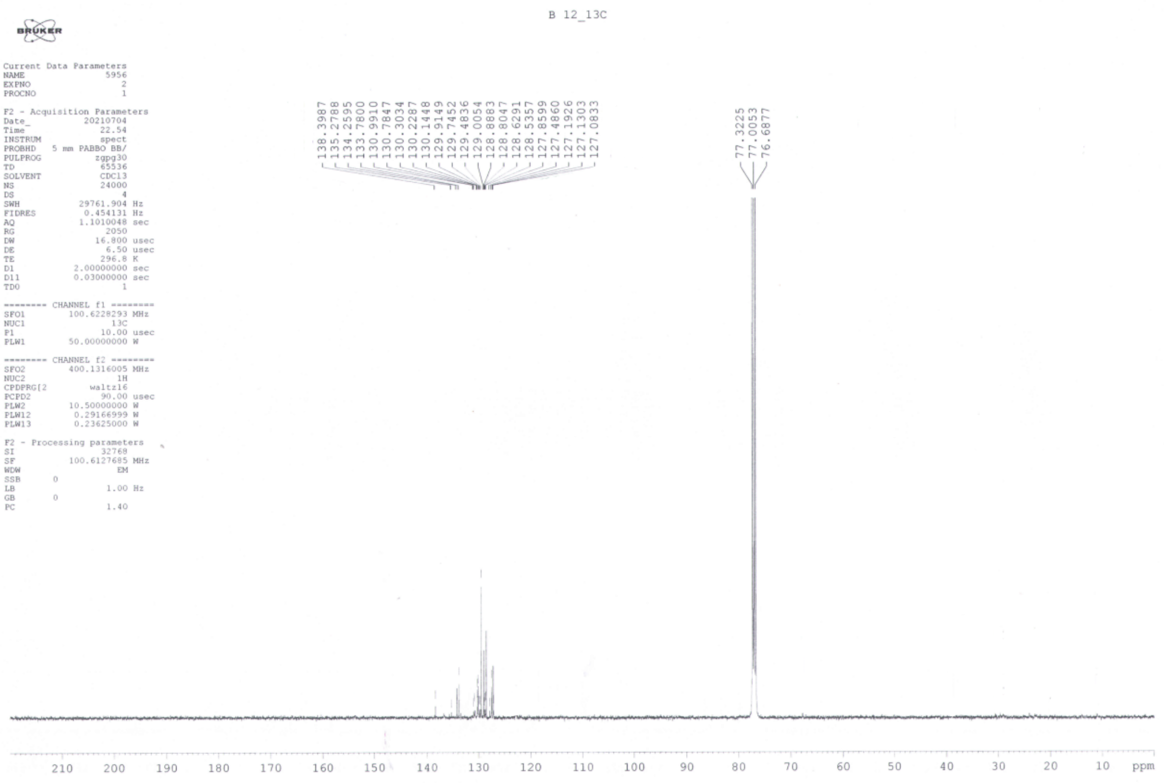

Enlarged spectrum in the range of 110-140 ppm.

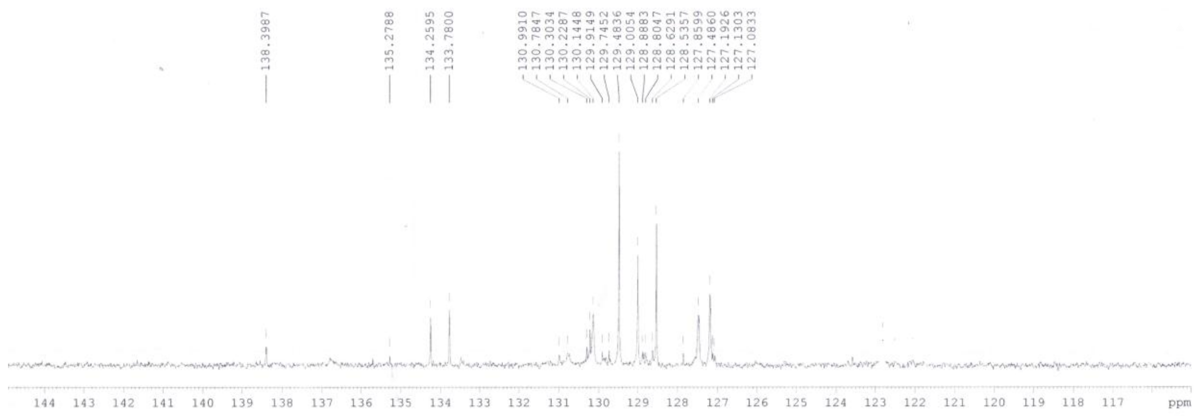

^{1}H spectrum of **AN7**

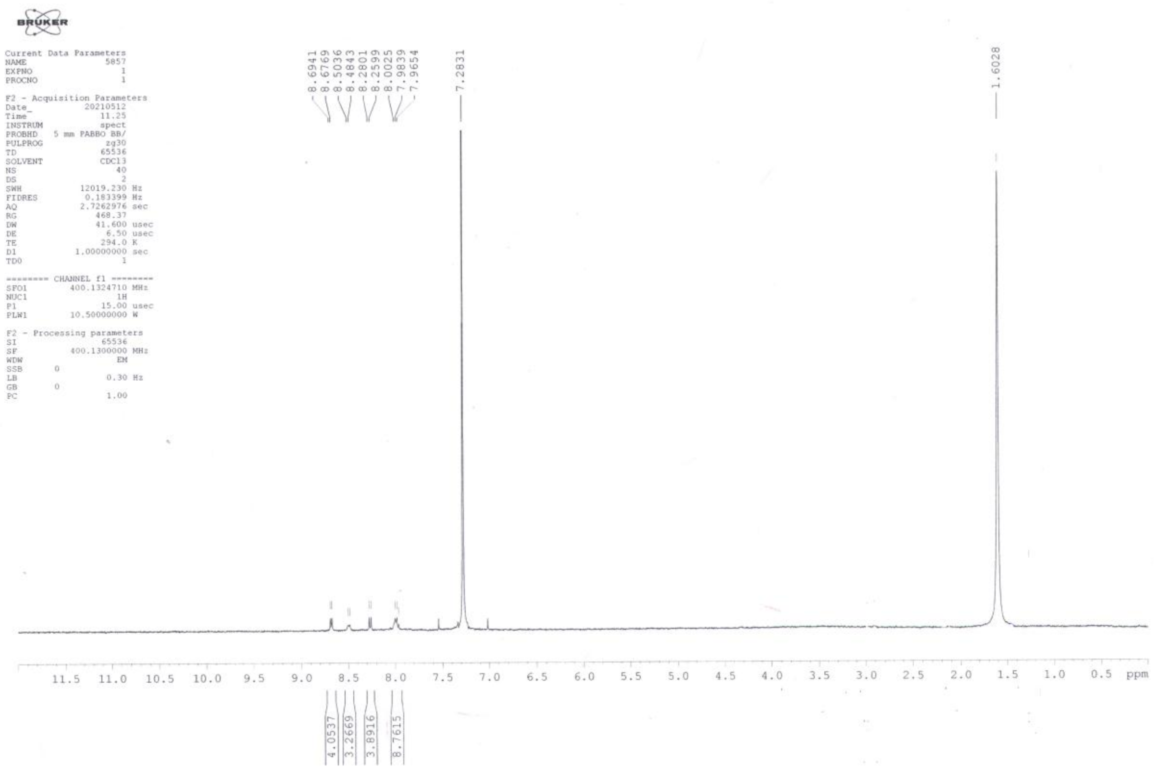

^{1}H spectrum of **AN8**

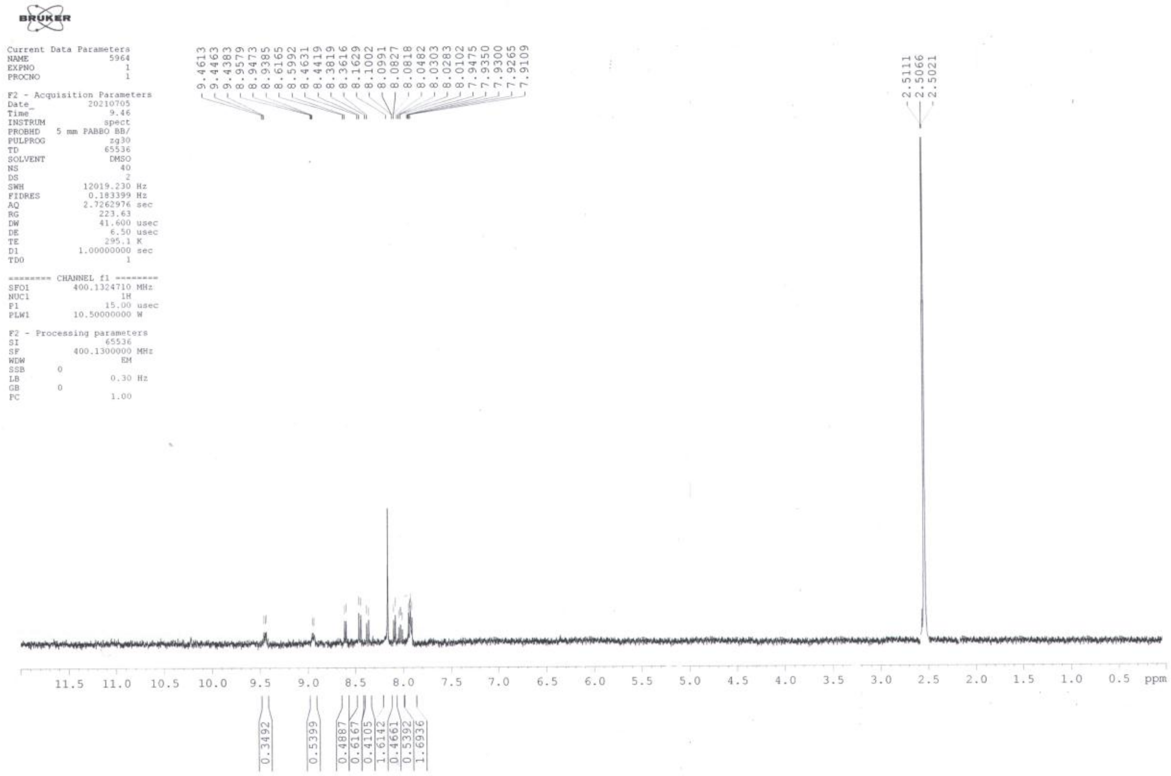

Enlarged spectrum in the range of 7-10 ppm.

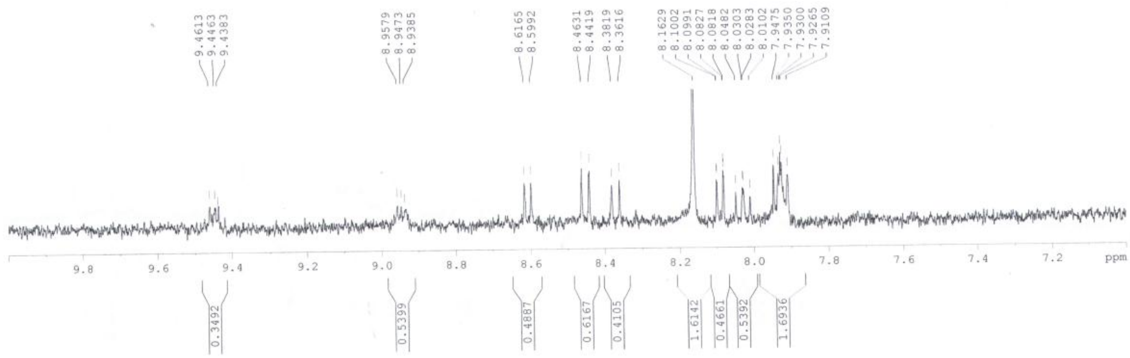

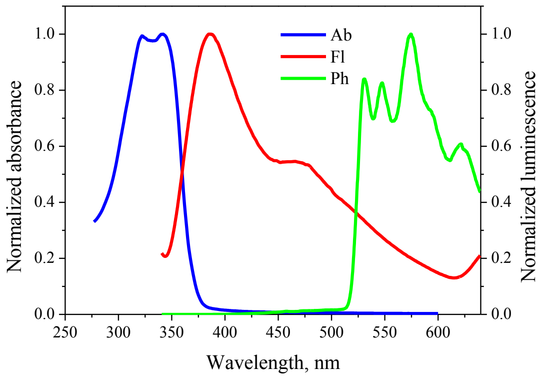

AN1

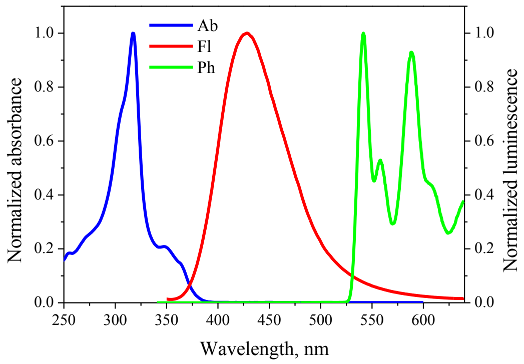

AN2

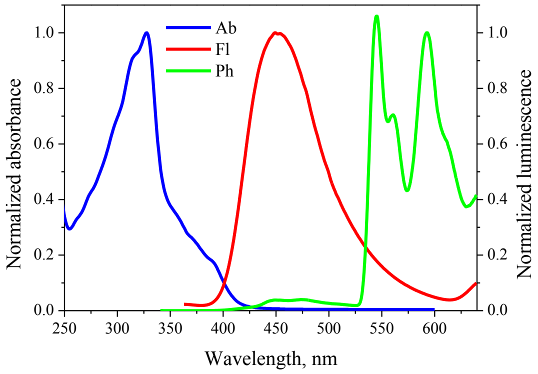

AN3

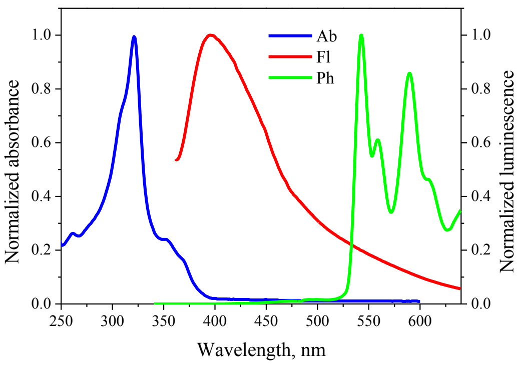

AN4

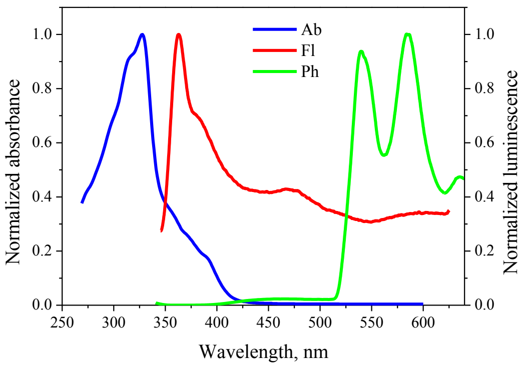

AN5

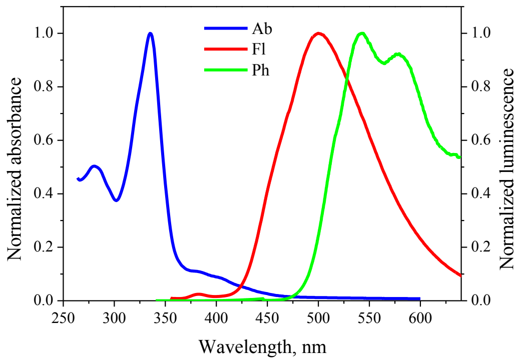

AN6

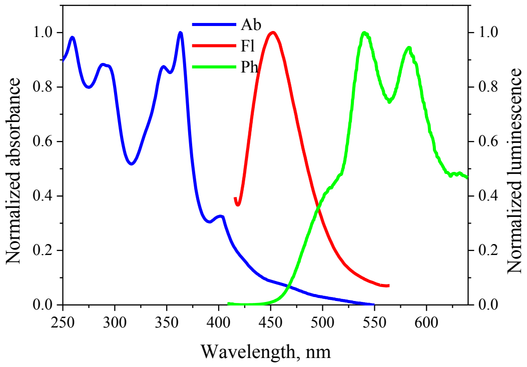

AN7

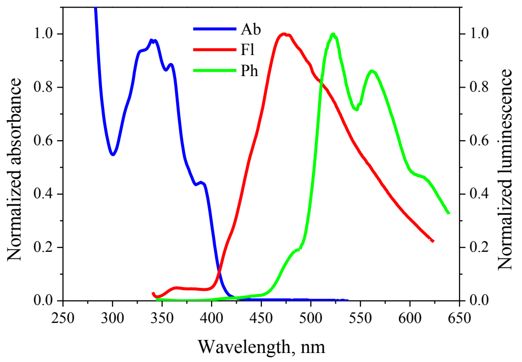

AN8

Figure S1. Normalized electronic absorption and emission spectra of acenaphthoquinoxaline derivatives in ethanol illustrating the influence of dye structure on the bands position.
